# Novel insights of waterborne human rotavirus A in Riyadh (Saudi Arabia) involving G2 predominance and emergence of a thermotolerant sequence

**DOI:** 10.1038/s41598-021-91607-3

**Published:** 2021-06-09

**Authors:** Islam Nour, Atif Hanif, Ibrahim O. Alanazi, Ibrahim Al-Ashkar, Abdulkarim Alhetheel, Saleh Eifan

**Affiliations:** 1 Botany and Microbiology Department, College of Science, King Saud University, Riyadh, Saudi Arabia; 2 National Center for Biotechnology, King Abdulaziz City for Science and Technology, Riyadh, Saudi Arabia; 3 Biotechnology Laboratory, Plant Production Department, College of Food and Agriculture Sciences, King Saud University, Riyadh, Saudi Arabia; 4 Agronomy Department, Faculty of Agriculture, Al-Azhar University, Cairo, Egypt; 5 Department of Pathology and Laboratory Medicine, College of Medicine, King Saud University, Riyadh, Saudi Arabia

**Keywords:** Molecular biology, Microbiology, Environmental microbiology, Virology

## Abstract

The routine evaluation of water environments is necessary to manage enteric virus-mediated fecal contamination and the possible emergence of novel variants. Here, we detected human rotavirus A (HRVA) circulating in two wastewater treatment plants, two lakes, irrigation water and a wastewater landfill located in Riyadh. VP7-derived surface protein sequences were assessed by phylogenetic analyses and inspection of thermotolerance-mediated secondary structure and seasonal variation. HRVA was most prevalent at An-Nazim wastewater landfill (AN-WWLF; 63.89%). Phylogenetic analyzes revealed the predominance of HRVA G2 lineage for the first time in Saudi Arabia. Moreover, a single HRVA sequence (2B64I-ANLF3/2018) was recovered at 45 °C from AN-WWLF; secondary structure prediction indicated that this sequence was thermotolerant with a high hydrophobicity, an absence of Ramachandran outliers, and a higher content of proline patches on the protein surface. Varied relationships were significantly observed between sampling areas influenced by temperature ranges (*p* < 0.05). HRVA prevalence was influenced by seasonal variations, favoring moderate temperatures in late autumn and early winter in all locations. However, a significant temperature impact was detected in Wadi-Hanifah Lake (*p* = 0.01). Our study extends the knowledge of currently circulating HRVA genotypes, and indicates the probable emergence of thermotolerant strains and seasonally mediated HRVA prevalence.

## Introduction

Wastewater (WW) is usually discharged to various surface water resources^1,2^. In addition to the negative impact of WW-mediated fecal contamination on aquatic environments, several public health issues are raised. Enteric viruses are a common cause of waterborne diseases associated with recreational water that were contaminated by the accidental release of feces or body fluids^3^. Moreover, their resistance to various environmental conditions has imposed a greater concern in terms of its durability and infectivity. Of a particular concern, rotavirus particles showed a significant tolerance to different pH, temperatures, electrolytes, etc.^4–6^. For instance, rotavirus can survive at a wide pH range (3–11) and for at least 7, 14, 22 and 32 days at 37 °C, 20 °C, 4 °C and − 20 °C, respectively with a slight loss of infectivity at 37 °C after 4 days^7,8^.

Rotaviruses are major causes of acute diarrhea in infants, leading to 9% mortality in the under-5 age group globally^9^. The high rotavirus incidence rate imposes a significant burden on both low- and high-income countries^10^. Rotavirus infection results in approximately 39% of diarrheal-mediated mortality, with a larger proportion occurring in developing countries^11^; most children will have experienced at least a single episode of rotavirus infection before they are 5 years old^12^. However, mortality rate due to rotavirus infection in < 5 year-old children has declined worldwide^13^. This could be owing to natural rotavirus infection mediated acquired immunity^14^ or vaccine introduction^15^.

Rotavirus is a non-enveloped, double-stranded RNA virus with a triple layered icosahedral capsid^16^. Six structural viral proteins (VP1-VP4, VP6, and VP7) and six functional proteins (NSP1-NSP6) which are associated with rotavirus virulence^17^. Moreover, two surface proteins, including the glycosylated VP7 (G type) and the protease-sensitive VP4 proteins (P type), constitute the basis for dual classification-dependent rotavirus genotyping^18^. In addition, several G and P type combinations have been detected as their corresponding genes were discretely passed to progeny viruses via reassortment. So far, 36 G and 51 P types demonstrate intratypic variation^19^. The G1, G2, and G9 and the P^1^, P^4^, and P^8^ genotypes have been frequently recorded at high prevalence in Saudi Arabia and its surrounding countries^20–23^. Moreover, fluctuations in rotavirus G-P type combinations are influenced by seasonal variations and geographical locations^19^. Interestingly, VP7 protein molecules exists on virion surface in larger concentrations of about 6.5 times than that of VP4^24^. Consequently, VP7-based monoclonal antibodies (mAbs) possess higher viral neutralizing capacity than VP4-mAbs^25^. Moreover, the genetic variation and mutation rate of VP7 gene is significantly high which even influences primers used causing genotyping failure^26^. Therefore, continuous monitoring of the circulating G lineage could enhance the vaccination program efficacy. However, few studies were specifically performed on the circulating waterborne rotaviruses in Saudi Arabia.

The current study aimed to detect the circulating human rotavirus A (HRVA) in six distinct locations in Riyadh, the capital of Saudi Arabia, including two WW treatment plants, two major lakes, irrigation water and a WW landfill. Moreover, the VP7-derived sequences from the HRVA detected were subjected to phylogenetic analysis. As Saudi Arabia is characterized by extreme heat^23^, the secondary structure of the detected sequences was investigated to justify probable differences in viral tolerance to higher temperatures. Additionally, the seasonal variation was inspected in the context of HRVA incidence in these sampling areas.

## Methods

### Sample collection

To monitor the HRVA in different water environments in Riyadh, samples were collected from two wastewater treatment plants (WWTPs): the KSU-WWTP and Manfouhah (MAN)-WWTP (also called the Southern Plant of Riyadh Wastewater Treatment Plant), two lakes (Wadi-Hanifah and Wadi-Namar), KSU irrigation treated water (IRTW) and a WW landfill of Riyadh eastern region (An-Nazim landfill [AN-WWLF]) (Fig. S1). Moreover, KSU-WWTP-dependent irrigation water was monitored for enteric viruses. The KSU-WWTP lies to the north of Riyadh and is designed with an average capacity of 9100 m^3^ day^−1^ and an average flow rate of 9000 m^3^ day^−1^. Primary treatment, involving pre-aeration, bar screen, grit chamber, comminutors, and primary sedimentation, are applied together with secondary treatment, comprising trickling filters and final sedimentation^27^. The KSU-WWTP is used for the irrigation of landscapes and for cooling purposes in the KSU power plant. The MAN-WWTP is south of Riyadh and was established with an average capacity of 200,000 m^3^ day^−1^ and a maximum flow rate of 250–320 m^3^ day^−1^^27,28^. Mechanical screens, pre-aeration, and primary sedimentation are implemented for primary treatment, followed by high-rate trickling filters and humus tanks for secondary treatment, and eventually, 52 sand filters for advanced treatment. The MAN-WWTP is utilized for industrial needs, restricted irrigation, and sewer flushing. Furthermore, Wadi Hanifah lake (WHL) includes the mainstream that extends for 120 km along Riyadh from the Al Uyaynah at the north of to Al-Hair city at the south^29^. Wadi-Namar lake (WNL) is one of the secondary valleys that branch from Wadi-Hanifah and includes the drainage of the Riyadh western area. This was used previously as a water source, but is currently used for partial WW disposal^30^. Water samples (1L each) were collected using 2-L low density polyethylene bottle (BRAND, Merck KGaA, Darmstadt, Germany) from WWTPs effluents, mid-stream of lakes (at a depth of about 30 cm), landscape flushing system endpoints (IRTW), WW landfill drainage endpoints (AN-WWLF). Water bottles were stored in a cooler box and transferred to the lab for processing within 2 h to avoid samples distortion and incidence of false negatives. Water samples were collected thrice every month for 1 year from April 2018 to March 2019 from each sampling area to check the seasonal influence on the HRVA prevalence. Moreover, both high and low temperatures were recorded at each collection day, obtained from accuweather website (https://www.accuweather.com/en/sa/riyadh). Water pH was measured by the electrode probe method using Orion Star A111 pH meter (Thermo Scientific Co., USA) and adjusted to neutral pH prior to viral concentration.

### Viral concentration

Rotavirus A from water samples was recovered using the electronegative-charged membrane-based adsorption/elution concentration method with a recovery efficiency of 56%^31^. To begin with, a HA filter of 0.45 µm pore size (Sigma-Aldrich Co., St. Louis, MO, USA) complying with U.S. EPA Standard methods was used to filter 5-mL aliquots of 0.25 M AlCl_3_. Each water sample (1 L) was then filtered through the Al^3+^-coated filter. The filter membrane was then flushed with 200 mL H_2_SO_4_ (0.5 mM) at pH 3. Subsequently, rotavirus A was eluted with 10 mL NaOH (1 mM). The eluent was neutralized with 100 µL equal volumes of H_2_SO_4_ (50 mM) and 100 × Tris–EDTA buffer (pH 8.0). The final volume reduction to 700 µL was performed using Amicon Ultra-15 (Merck Millipore Ltd., Carrigtwohill Co., Cork, Ireland) for achieving > 10^3^ ×, followed by storage at − 80 °C.

### Nucleic acid extraction and RT-PCR

HRVA RNA was extracted from 200 µL of the viral concentrates using a ZymoBIOMICS RNA Mini Kit (Zymo Research, Irvine, CA 92614, USA) following the manufacturer’s instructions. HRVA RNA was reverse transcribed using Sensiscript Reverse Transcription (RT) Kit (SensRT; Qiagen GmbH, Hilden, Germany). RT was performed in a 20-µL reaction mixture consisting of 2 µL template RNA and 18 µL RT mixture containing 2 µL 10 × RT buffer, 2 µL dNTPs, 2 µL random hexamers (final concentration of 10 μM), 1 µL Sensiscript RT, 1 µL RNase inhibitor (40 U µL^-1^), and 10 µL nuclease-free water. The RT mixture was incubated at 37 °C for 1 h. RT was followed by polymerase chain reaction (PCR) using the 2 × Phusion Master Mix with GC buffer (Thermo Scientific, New England Biolabs, Ipswich, MA 01,938, USA). PCR was performed in a 20-µL reaction mixture consisting of 2 µL cDNA template, RComb-F: 5′-CCACAAYTDTTGTGATTA-3′, RComb-R 5′-CCCATYGTATCCAYTTATT-3′ (500 nM each) (designed for targeting 173 bp, located at 313–485 nucleotides in VP7, Reference strain: BLU5, Accession number: LC500701), and the 1 × Phusion Master Mix under the following reaction conditions: 98 °C for 30 s, followed by 40 cycles of 98 °C for 10 s, 50 °C for 30 s, and 72 °C for 30 s each, and a final extension at 72 °C for 5 min. A HRVA-positive control was obtained from the Virology Unit, King Khalid University Hospital, Riyadh, Saudi Arabia.

### Amplicon purification and sequencing

PCR products were visualized by 2 × agarose gel electrophoresis and 173-bp amplicons were excised and purified using the Wizard SV Gel and PCR Clean-Up System (Promega Co., Madison, WI, USA), following the manufacturer’s instructions. The purified amplicons were sequenced in both forward and reverse directions (3X) using the BigDye Terminator v3.1 cycle sequencing kit (Applied Biosystems, Foster City, California, USA) with individual PCR primers yielding the second round PCR product. Sequences were obtained using an ABI genetic analyzer 3130Xl (Applied Biosystems, Carlsbad, California 92008. USA).

### Phylogenetic analysis

The sequences obtained were cleaned by removing the overlapping sequences after comparison with the sequences obtained from paired-end sequencing using Bioedit v7.2 (Nucleics Co., Sydney, Australia). To compare nucleotide sequences obtained from water samples, phylogenetic analyses were performed for the resulting HRVA sequences against the HRVA G1 lineage represented by G1P[8], G9 lineage, including G9P[8], G2 lineage, including G2P[4] and G2P[8], as the later genotypes were reported as highly prevalent in Saudi Arabia and nearby countries^20–23^. The G12 lineage was designated as the outgroup. The sequence alignments were generated using the multiple sequence alignment tool ClustalW in MEGA X software^32^, with an opening penalty of 15 and an extension penalty of 6.66. Subsequently, the aligned sequences were assessed for the best fitting substitution model to construct the phylogenetic tree on analysis. The models with the lowest Bayesian information criterion were considered as the best depiction of the substitution pattern. Moreover, the Akaike information criterion, corrected value, maximum likelihood value (*lnL*), and the count of parameters used (including branch lengths) were considered for each model^33^. The non-uniformity of evolutionary rates among sites was modeled by applying a discrete Gamma distribution (+ *G*) with five rate categories and assuming that a certain fraction of sites is evolutionarily invariable (+ *I*). Furthermore, tree topology was automatically computed to estimate ML values. A phylogenetic tree was constructed using aligned sequences by the neighbor-joining method implementing 1000 bootstrap replicates using MEGA X software^32^. This analysis involved 94 nucleotide sequences. The genetic distances were estimated by the Kimura three-parameter method.

### Secondary structure investigation

The influence of the secondary structure of the detected HRVA sequence (located at amino acids 105–161 of VP7 of the reference strain BLU5, Accession number: BBN91702) on thermotolerance, including any possible deviation from mesophilic-detected HRVA isolates, was assessed using SWISS-MODEL (https://swissmodel.expasy.org/)^34^. Moreover, the detection and orientation of distinctive residues and the abundance of possible residual outliers were investigated using Ramachandran plotting and MolProbity^35^. The sequences of 2B64I-ANLF3/2018 and 2B64I-ANLF5/2018 (target) were modeled against the structure of the VP7 outer layer protein using an atomic model of an infectious rotavirus particle (protein databank [PDB]:4V7Q.1.U) as a template. Furthermore, the models obtained for both 2B64I-ANLF3/2018 and 2B64I-ANLF5/2018 were used for alignment and comparison via the CLC Main Workbench V20.0 (Qiagen).

### Statistical analyses

Pearson’s correlation coefficient matrix was implemented to define the probable relationship between the sampling areas over the 1-year period. Moreover, the influence of seasonal variation, denoted by a wide range of high and low temperatures, on HRV detection was examined. One-way analysis of variance was performed to test the influence and significance of high- and low-temperature ranges on HRVA incidence, regardless of the sampling area. The relationships between different sampling areas (as dependent variables) and high and low temperatures (as independent variables) were fitted using linear curve-fitting. All statistical analyses were conducted using the XL-STAT statistical package software (Ver. 2019, Excel Add-ins soft SARL, New York, NY, USA).

## Results

### AN-WWLF prevalence over other sampling areas

Out of 216 water samples tested for HRVA, 53 (24.5%) were positive as denoted by the detection of the 173-bp amplicon. The highest HRVA prevalence (approximately 64%) was observed in the AN-WWLF, whereas the lowest prevalence occurred in the MAN-WWTP and IRTW (approximately 6%) (Table 1). The molecular characterization of amplicon sequences by Sanger sequencing showed the presence of 35 sequences; however, 18 sequences were not characterized owing to overlapped electropherograms, indicating the presence of more than one HRVA type.

**Table 1:** HRVA prevalence in different sample locations.

Sampling area	HRVA + ve 2018	HRVA + ve 2019	HRVA prevalence (%)
KSU-WWTP	3	2	13.89
MAN-WWTP	2	0	5.56
WHL	7	3	27.78
WNL	8	3	30.56
AN-WWLF	16	7	63.89
IRTW	1	1	5.56

### Predominance of lineage G2 of HRVA

**Figure 1:**
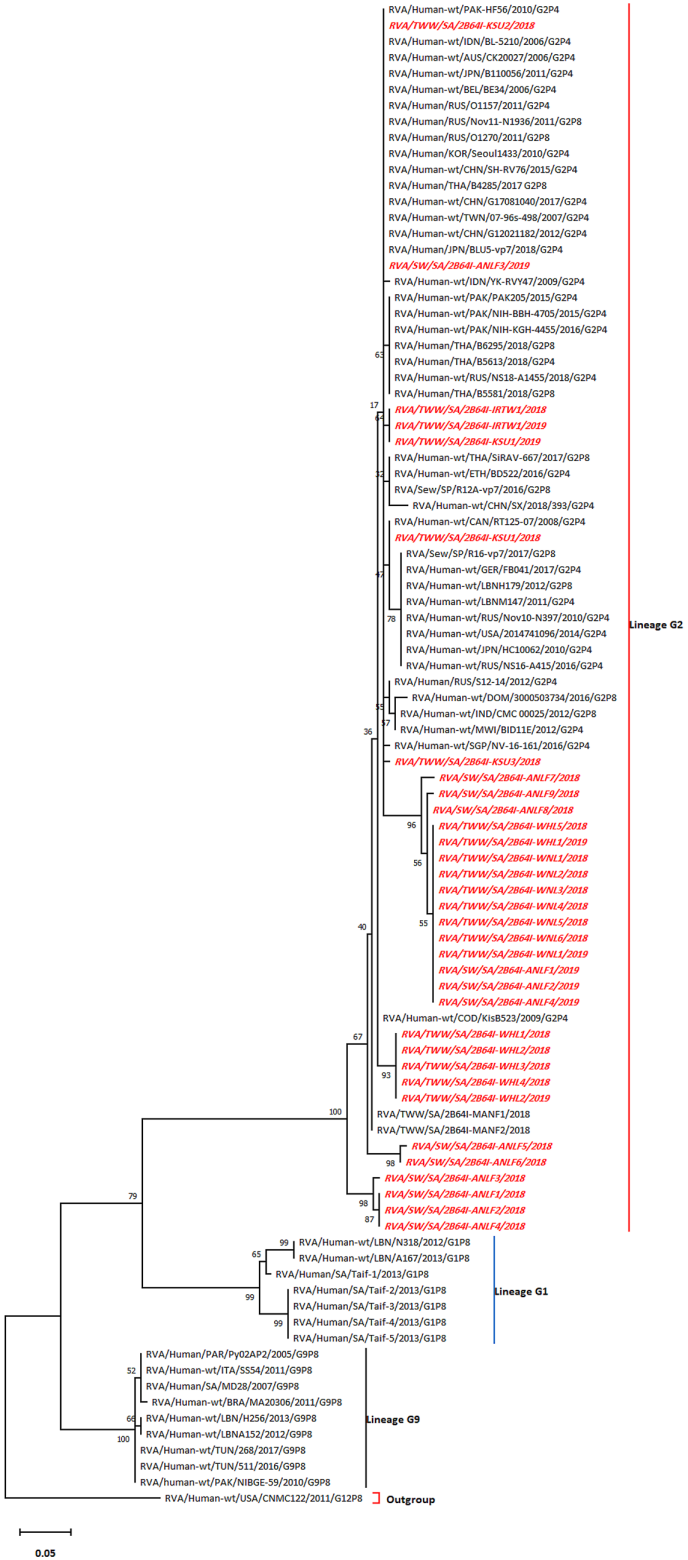
Phylogenetic tree for the HRVA VP7-derived sequences constructed by the maximum likelihood method and Tamura three-parameter model. The highest log likelihood tree is displayed (− 898.28). The percentage of associated taxa that are clustered together are provided at each branch. Heuristic search-dependent initial trees were produced automatically via Neighbor-Join and BioNJ algorithms applied to the pairwise distances matrix and assessed by the maximum composite likelihood (MCL) approach, followed by the highest log likelihood-resulted topology selection. The rate variation model was permitted to be evolutionarily invariable for several sites ([+ I], 44.78% sites), according to the best fitting substitution model validation (Table S2). The horizontal distance connecting two HRVA sequences is proportional to the genetic distance between these two HRVA sequences. The distance is expressed as the number of the nucleotide substitutions per site. Strain CNMC122 (G12P[8]) was used as an outgroup. Accession numbers of sequences used for phylogenetic analysis are displayed in Table S3.

The phylogenetic tree displayed a clear relationship between these HRVA sequences and the G2 lineage rather than the G1 and G9 lineages, as the current study sequences were entirely in the G2 lineage (Fig. 1). Pairwise distancing uncovered the presence of eight HRVA isolates of null distance to other isolates and/or strains (Tables 2 and S1). Initially, IRTW-derived HRVA sequences were related closely to HRVA sequences isolated from the KSU-WWTP obtained in 2019 (2B64-KSU1). However, another KSU-WWTP-derived HRVA sequence detected in 2018 (2B64-KSU1) was related to an HRVA Canadian strain (RT125-07). Moreover, sequences recovered from the MAN-WWTP were more linked to each other than to isolates from other sources. Remarkably, the HRVA sequence obtained from the KSU-WWTP (2B64I-KSU2/2018) was typically related to several genotypes G2P[4] and G2P[8] from Asia, Australia, and Europe. However, another AN-WWLF isolate (2B64-ANLF1/2019) was more related to isolates obtained from WHL, WNL, and AN-WWLF rather than to strains from other countries. Moreover, the constructed tree depicted two closely related AN-WWLF sequences (2B64-ANLF5/2018 and 2B64-ANLF6/2018), which were relatively distant to another cluster of AN-WWLF sequences (2B64-ANLF1–4 in 2018). Interestingly, the latter cluster comprised a sequence of an HRVA isolate (2B64-ANLF3/2018) recovered at a relatively high temperature (45 °C), unlike the HRVA sequences (2B64-ANLF5/2018 and 2B64-ANLF6/2018) in the former branch obtained at lower temperatures (22 °C and 23 °C, respectively). Therefore, the secondary structure of 2B64-ANLF3/2018 was compared with those obtained at the lowest temperature because of the absence of any sequence obtained from other sampling areas at 45 °C.

**Table 2:** HRVA sequences grouping (Grouping is based on zero-distance between studied sequences as well as extraneously with other strains used for phylogenetic analysis implemented by pairwise distancing using Kimura three-parameter method).

Studied sequence	Similar sequence(s)	Country of origin*
2B64I-KSU1/2018	RT125-07/2008/G2P4	Canada
2B64I-MANF1/2018	2B64I-MANF2/2018	Saudi Arabia
2B64I-WHL1/2018	2B64I-WHL2/2018	
	2B64I-WHL3/2018	
	2B64I-WHL4/2018	
2B64I-WHL1/2019	2B64I-WHL2/2018	
	2B64I-WHL3/2018	
	2B64I-WHL4/2018	
2B64I-ANLF1/2019	2B64I-WHL1/2019	Saudi Arabia
	2B64I-WHL5/2018	
	2B64I-WNL1/2018	
	2B64I-WNL2/2018	
	2B64I-WNL3/2018	
	2B64I-WNL4/2018	
	2B64I-WNL5/2018	
	2B64I-WNL6/2018	
	2B64I-WNL1/2019	
	2B64I-ANLF2/2019	
	2B64I-ANLF4/2019	
2B64I-ANLF1/2018	2B64I-ANLF2/2018	Saudi Arabia
	2B64I-ANLF4/2018	
2B64I-KSU2/2018	BLU5-vp7/2018/G2P4	Japan
	G12021182/2012/G2P4	China
	07-96s-498/2007/G2P4	Taiwan
	G17081040/2017/G2P4	China
	B4285/2017/G2P8	Thailand
	SH-RV76/2015/G2P4	China
	Seoul1433/2010/G2P4	South Korea
	O1270/2011/G2P8	Russia
	Nov11-N1936/2011/G2P8	Russia
	O1157/2011/G2P4	Russia
	BE34/2006/G2P4	Belgium
	B110056/2011/G2P4	Japan
	CK20027/2006/G2P4	Australia
	BL-5210/2006/G2P4	Indonesia
	PAK-HF56/2010/G2P4	Pakistan
	2B64I-ANLF3/2019	Saudi Arabia
2B64I-IRTW1/2018	2B64I-KSU1/2019	Saudi Arabia
	2B64I-IRTW1/2019	

*Country of origin of similar sequences.

### Thermotolerance indicated by predicted secondary structure hydrophobicity and fewer Ramachandran outliers of HRVA 2B64I-ANLF3/2018 isolate

**Figure 2:**
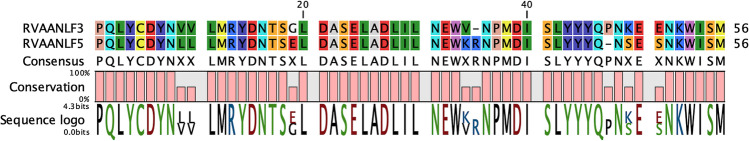
Amino acid sequence alignment of 2B64I-ANLF3/2018 (designated RVAANLF3) and 2B64I-ANLF5/2018 (designated RVAANLF5).

**Figure 3:**
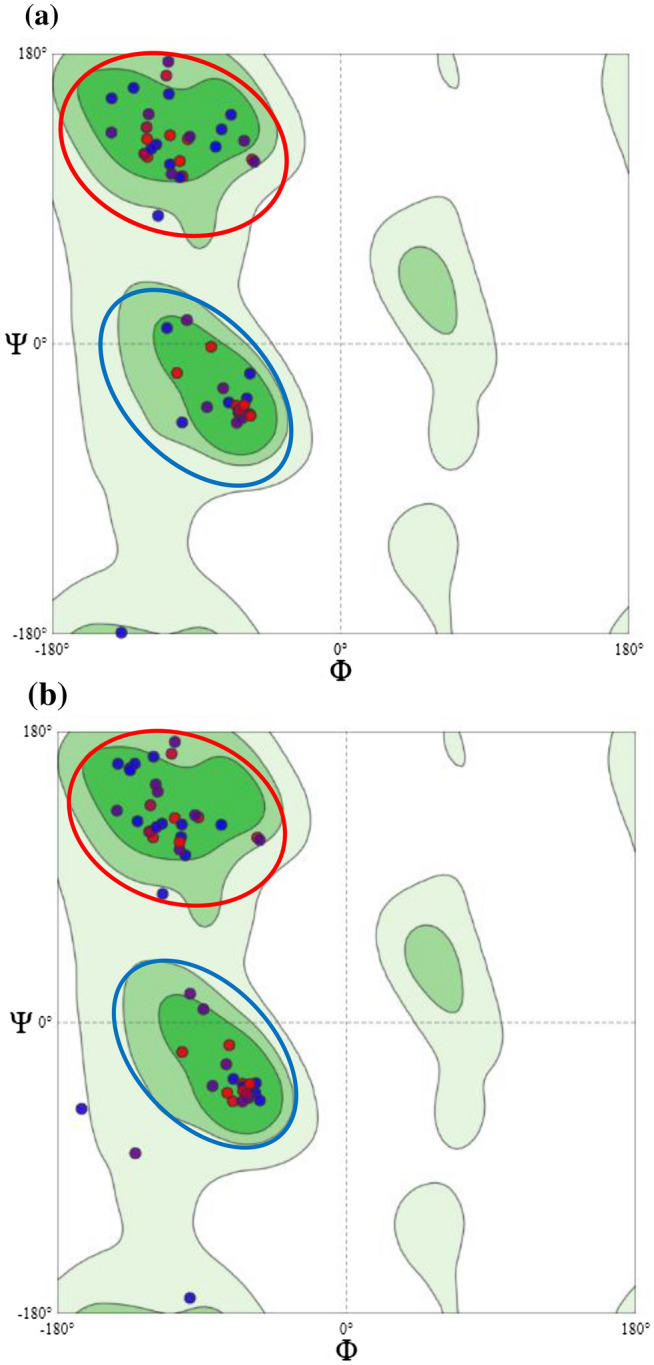
Ramachandran plot showing hydrophobic residues for (**a**) 2B64I-ANLF3/2018 and (**b**) 2B64I-ANLF5/2018. Ramachandran plot was obtained by using SWISS-MODEL (https://swissmodel.expasy.org/). The red circle refers to hydrophobic residues in the β-sheet. The blue circle denotes the hydrophobic residues in right-handed helix. Residues outside the circles represent residual outliers.

The alignment of amino acid sequences identified a shared identity for both 2B64I-ANLF5/2018 and 2B64I-ANLF6/2018; therefore, 2B64I-ANLF5/2018 was used as a representative for further analyses. Initially, the alignment of the predicted amino acids sequences showed the substitution of valine (a non-polar amino acid) in 2B64I-ANLF3/2018 by lysine (a polar amino acid) in 2B64I-ANLF5/2018 at codon 34 (Fig. 2). Moreover, the proline content of 2B64I-ANLF3/2018 (Movie 1) was higher than that of 2B64I-ANLF5/2018 (Movie 2) that patched over the external surface, mediating structural stability. Furthermore, outliers were absent in the Ramachandran plot for 2B64I-ANLF3/2018, and 94.44% of the constructed structure was in the favorable region of the plot, whereas 2B64I-ANLF5/2018 had 1.85% outliers and 88.89% of the structure in the favored region (Fig. 3). In addition, a three-dimensional secondary structure alignment showed that the substitution of valine and lysine in 2B64I-ANLF3/2018 with leucine and serine residues in 2B64I-ANLF5/2018 (Fig. 2) resulted in an initial shift in the β-sheet at codon 9, which then affected the structural alignment between codons 29 and 33 and a looped distance at codon 49 (Fig. S2). However, the substitution of glycine (2B64I-ANLF3/2018) by glutamic acid (2B64I-ANLF5/2018) at the base of an α-helix had no effect on the structural alignment. In addition, both sequences contained an N-glycosylation site (NTS**G** for 2B64I-ANLF3/2018 and NTS**E** for 2B64I-ANLF3/2018; however, 2B64I-ANLF5/2018 was shown to possess an extra protein kinase C phosphorylation (PKCP) site (SNK) (Fig. S3). This combination of structural features indicates that this isolate can tolerate higher temperatures, unlike other HRVA isolates. Moreover, this sequence was not detected in any other sampling area. Therefore, the relationship between the sampling area and the impact of seasonal variation on HRVA incidence was further investigated.

### Relationship differences between sampling areas

The relationships detected between the studied sampling areas at high temperatures (the numbers above the gray highlight, Table 3) differed from those obtained at low temperatures (the numbers below the gray highlight, Table 3). At high-temperature range, IRTW was of the highest significant correlation (*p* = 0.012) to the KSU-WWTP, followed by a slightly lower correlation of AN-WWLF to WHL and WNL (*p* = 0.02 and 0.021, respectively) and WNL to WHL (*p* = 0.037) in terms of the % HRVA detection. On the contrary, at the low-temperature range, IRTW was of the highest correlation to AN-WWLF (*p* = 0.002) followed by a significant correlation of WHL to AN-WWLF, IRTW, and WNL (*p* = 0.003, 0.005, and 0.014, respectively) and KSU-WWTP to WNL and WHL (*p* = 0.006 and 0.013, respectively).

**Table 3:** Pearson’s correlation matrix of HRVA detection percentage at the various sampling areas.

	% Det_KSU-WWTP_	% Det_MAN-WWTP_	% Det_WHL_	% Det_WNL_	% Det_AN-WWLF_	%Det_IRTW_
% Det_KSU-WWTP_		0.773	0.796	0.627	0.637	0.908*
% Det_MAN-WWTP_	0.686		0.347	0.423	0.196	0.632
% Det_WHL_	**0.905**	0.631		**0.838**	**0.882**	0.614
% Det_WNL_	**0.935**	0.466	**0.900**		**0.881**	0.267
% Det_AN-WWLF_	0.751	0.537	**0.957**	0.786		0.402
% Det_IRTW_	0.759	0.632	**0.943**	0.799	**0.967**	

The numbers above the grey-highlighted diagonal refers to correlation values at high-temperature range, whereas correlation values at low-temperature range are below the diagonal. % Det denotes HRVA detectability percentage at different sampling areas, for instance % Det_KSU-WWTP_ refers to HRVA detection percentage at KSU-WWTP.

*Significant correlation values are displayed as bold numbers.

### Seasonal variation influenced HRVA incidence

**Figure 4:**
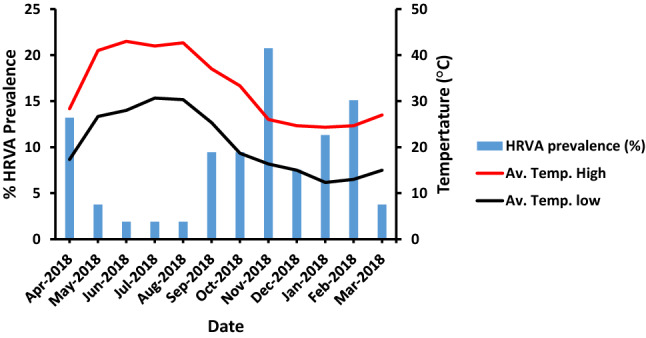
Temperature variation influence on the HRVA prevalence in water samples. “Av. Temp. High” refers to the average high temperature and “Av. Temp. Low” refers to the average low temperature.

Regardless of the sampling area, the incidence of HRVA was significantly affected by temperature variations (*p* = 0.03), with a higher prevalence at lower temperatures (Fig. 3). However, when the sampling areas were segregated, HRVA was detected mostly at moderate temperature ranges (26 °C–29 °C) at five sampling areas in the high-temperature range, unlike WNL that favored a lower temperature range (22 °C–25 °C) (Fig. 4). HRVA detection followed a similar pattern at the low-temperature range of 14 °C–17 °C, which roughly corresponded to that at 26 °C–29 °C in the high-temperature range, thus favoring late autumn and early winter seasons (Fig. S3). Strikingly, HRVA was detected in the AN-WWLF at both high and low temperatures, unlike the other sampling areas. Despite the existence of clustered HRVA at relatively low temperatures in all sampling areas, a significant influence of the high-temperature range on HRVA existence was detected only at WHL (*p* = 0.01) (Table 4).

**Table 4:** The significance of impact of high or low-temperature ranges on HRVA prevalence in various sampling areas.

Sampling area	Temperature range	R^2^	RMSE	Equation
KSU-WWTP	High	0.353	21.022	% Prev_KSU-WWTP_ = 74.24–1.86* T_H_^‡^
	Low	0.61	10.511	% Prev_KSU-WWTP_ = 46.52–1.57* T_L_
MAN-WWTP	High	0.043	28.242	% Prev_MAN-WWTP_ = 38.81–0.71* T_H_
	Low	0.043	28.242	% Prev_MAN-WWTP_ = 30.24–0.71* T_L_
WHL	High	0.844*	8.223	% Prev_WHL_ = 87.52–2.29* T_H_
	Low	0.442	18.797	% Prev_WHL_ = 54.67–2* T_L_
WNL	High	0.601	13.715	% Prev_WNL_ = 79.07–2.01* T_H_
	Low	0.553	13.189	% Prev_WNL_ = 49.98–1.75* T_L_
AN-WWLF	High	0.502	9.051	% Prev_AN-WWLF_ = 50.36–1.09* T_H_
	Low	0.281	12.88	% Prev_AN-WWLF_ = 34.96–0.96* T_L_
IRTW	High	0.154	41.975	% Prev_IRTW_ = 83.096–2.14* T_H_
	Low	0.154	41.975	% Prev_IRTW_ = 57.38–2.14* T_L_

% Prev refers to HAdV prevalence percentage at different sampling areas. RMSE denotes the root mean squared error which is an absolute measure of fit.

*Significant at *p* < 0.05.

^‡^T_H_ represents the highest temperature detected whereas T_L_ denoted lowest temperature detected.

## Discussion

Enteric viruses, particularly the non-enveloped viruses, are highly stable in water environments and fomite^36^, leading to potential viral dissemination^37^. Likewise, the current study addressed the detection of HRVA in WWTPs, surface waters, and a landfill. HRVA prevalence in AN-WWLP was similar to the previously reported rotavirus prevalence of 65% at the Al-Misk Lake in Jeddah, Saudi Arabia^38^. However, we detected a higher prevalence of HRVA in both lakes (around 27–31%) than that recovered from the Biljurashi dams and Mikhwah sites (~ 13%) in the Albaha region, Saudi Arabia^39^. The current higher prevalence of HRVA might be owing to the 1-year sampling plan in comparison with the 3-month period of the earlier study^39^. HRVA prevalence in the MAN-WWTP was less than that observed in the KSU-WWTP, which may be because of the additional advanced treatment phase following the secondary water treatment that is absent in the KSU-WWTP^27^.

Phylogenetic analyses of the HRVA detected in this study lay entirely within the G2 lineage. However, as reported by several studies in Saudi Arabia^22,23,40^, Lebanon^21^, Bahrain^41^, Kuwait^42^, and Oman^43^, the predominant lineage of HRVA was G1, whereas G3 was the most prevalent genotype in Qatar^19^. A recent study highlighted a surge in G2 incidence in Saudi Arabia, particularly the G2P[4] genotype, occurring after vaccination, which may partially justify our results^39^. Thus, vaccination-mediated selection pressure may have favored the prevalence of G2 rather than G1 strains of the HRVA. However, the discrepancy with the Qatar findings could be because of the contribution of expats (~ 80%) of the community structure, leading to the dominance of the G3 genotype^44,45^. Moreover, the close relationship between sequences isolated from IRTW (2B64I-IRTW1/2018 and 2B64I-IRTW1/2019) and KSU-WWTP (2B64I-KSU1/2019) was found due to the fact that the source of irrigation water belongs to the KSU-WWTP effluent. Strikingly, two closely related AN-WWLF sequences (2B64-ANLF5/2018 and 2B64-ANLF6/2018) were found distantly related to the 2B64-ANLF3/2018 sequence, which belonged to the same source; however, the latter sequence was recovered at a relatively high temperature, thus postulating the existence of possible differences between secondary structure of these sequences.

The current study detected higher hydrophobicity in the 2B64-ANLF3/2018 sequence in comparison to low-temperature-prevailing sequences and this might have enhanced high-temperature resistance, as described elsewhere^46^. In addition, the molecular thermodynamics of thermophilic proteins is enhanced by the increased use of small non-polar amino acids (e.g., valine) in addition to greater hydrophobicity, which is in agreement with our study findings^47^. Moreover, a higher proline content was detected in the higher-temperature-prevailing sequence (Movie 1) and led to improved structural tolerance to such harsh conditions. Proline introduction resulted in reduction of steric hindrance and the unfolded state entropy besides its side chain cyclic structure that supported conformational rigidity required for thermotolerance^48,49^. Furthermore, thermophilic proteins were reported to have fewer average residues in Ramachandran plot with more condensed residues in the β-sheet and right- and left- handed helix core areas than those of a mesophilic counterpart, which is in line with our results^50^. Moreover, the N-glycosylation motif was detected in both sequences and was implicated in conferring protein stability and thermotolerance for many viruses, including the severe acute respiratory syndrome coronavirus 2^51,52^, influenza A virus^53^, and West Nile virus^54^. However, 2B64-ANLF3/2018 contains a glycine in the N-glycosylation motif (NTSG) instead of the glutamic acid usually favored in thermophilic proteins in the helix structure (Fig. S2)^50,55^. Moreover, phosphorylation at PKCP motif declined along with temperature elevation, as recorded for the brome mosaic virus^56^ indicating sensitivity of PKCP motif to high temperature which approves PKCP motif presence in the low-temperature favoring sequence.

In contrast, this thermotolerant sequence was absent in sequences isolated from other locations; thus, varied relationships between sampling areas were found. KSU-WWTP is the treated water source of IRTW and this imposed the greatest relationship between them. However, at the low-temperature range, IRTW was highly correlated to AN-WWLF, which could be because of the discharge of agricultural wastes to the AN-WWLF, although this reason is still doubtful because of the approximately 25-km distance between these sites that would make HRVA vulnerable and less likely to persist. Moreover, the significant relationship between the lakes is because of their geographical nature, as WNL is a subsidiary branch of WHL^30^.

Seasonal variation was found to influence the prevalence of HRVA, indicating that there was a higher prevalence at lower temperatures, which agrees with results of various studies that favored its prevalence in winter months^22,57,58^. Interestingly, significant HRVA prevalence was noted at the lower temperature range at WNL, which could be attributed to uncovered surface water exposed to various limiting conditions like direct sunlight, UV light, and high temperatures^59^. Moreover, the stable pattern of HRVA prevalence in AN-WWLF might be owing to HRVA sequence nature, fecal-mediated contamination from livestock, or contamination from vaccine strains^60^. Feces from vaccinated children younger than 6-month old were shown to boost HRVA prevalence in surface water^61^.

The current study provides knowledge regarding HRVA prevalence and seasonal influences in different water environments in Riyadh. The dominance of the G2 lineage was reported for the first time in Saudi Arabia. The emergence of an unexpectedly thermotolerant HRVA sequence at 45 °C demands the extensive monitoring of circulating strains in the future.

## Supplementary Information

Supplementary Information 1.

Supplementary Video 1.

Supplementary Video 2.
