# Supplementary material for: Novel insights of waterborne human rotavirus A in Riyadh (Saudi Arabia) involving G2 predominance and emergence of a thermotolerant sequence
Source: Sci Rep. 2021 Jun 9;11:12132. doi: 10.1038/s41598-021-91607-3 (PMC8190275; doi:10.1038/s41598-021-91607-3)
Supplement: Supplementary file 1 — Supplementary Information 1. [file 41598_2021_91607_MOESM1_ESM.docx]

**Supplementary information**

**Movie 1. Proline locations indicated in the 3D structure of the VP7-derived sequence of the 2B64I-ANLF3/2018 isolate.** Rotation movie of the predicted secondary structure is displayed in surface representation. Three proline residues are shown in blue, and one of them was present in a groove-like structure.

**Movie 2. Proline locations indicated in the 3D structure of the VP7-derived sequence of the 2B64I-ANLF5/2018 isolate.** Rotation movie of the predicted secondary structure is shown in surface representation. Two proline residues are displayed in blue, and one of them was present in a groove-like structure.

**
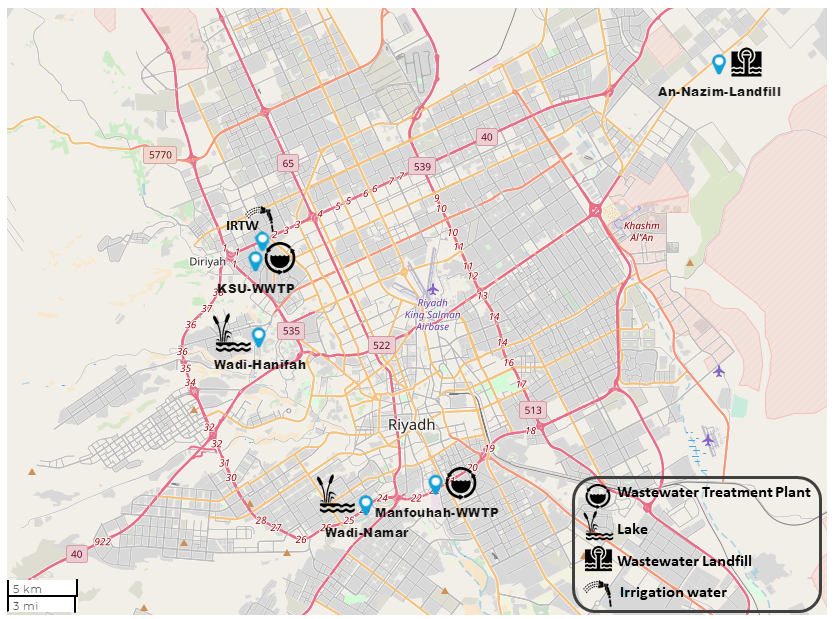
**

**Figure S1. Locations of sampling areas.**

The map was generated based on coordinates of sampling areas using Mapline integrated Excel Addin 2016 (https://app.mapline.com/map/map_4d55f49e).


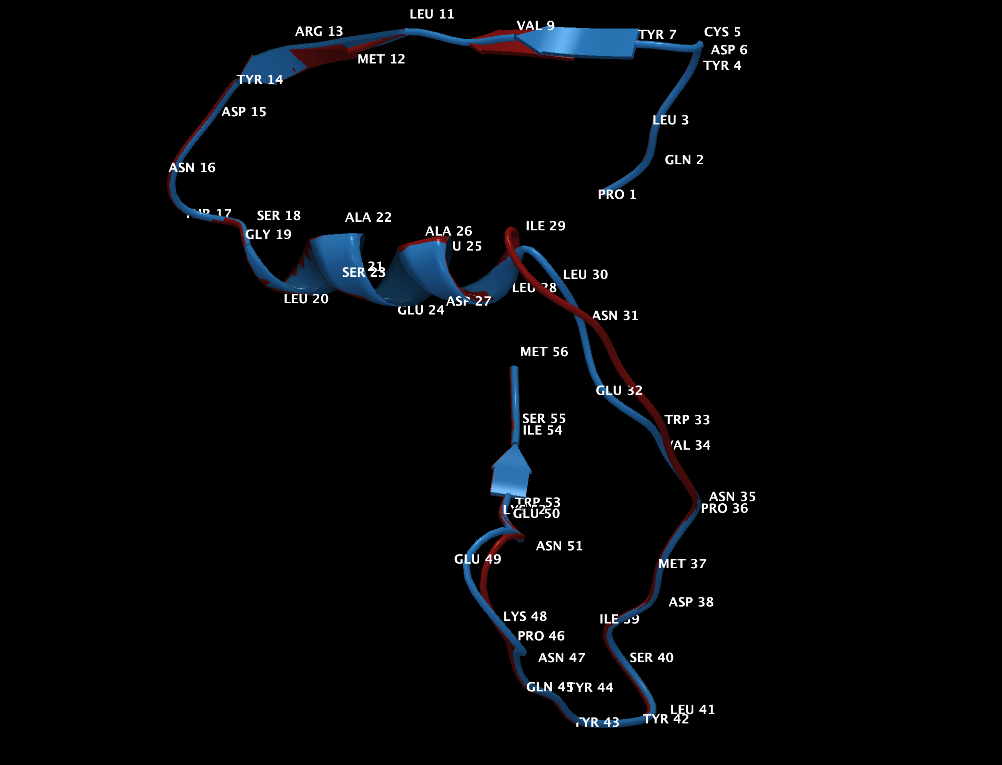


**Figure S2. 3D secondary structure alignment of VP7-derived sequences**. The blue strand refers to 2B64I-ANLF3/2018, whereas the dark red strand refers to 2B64I-ANLF5/2018.

**
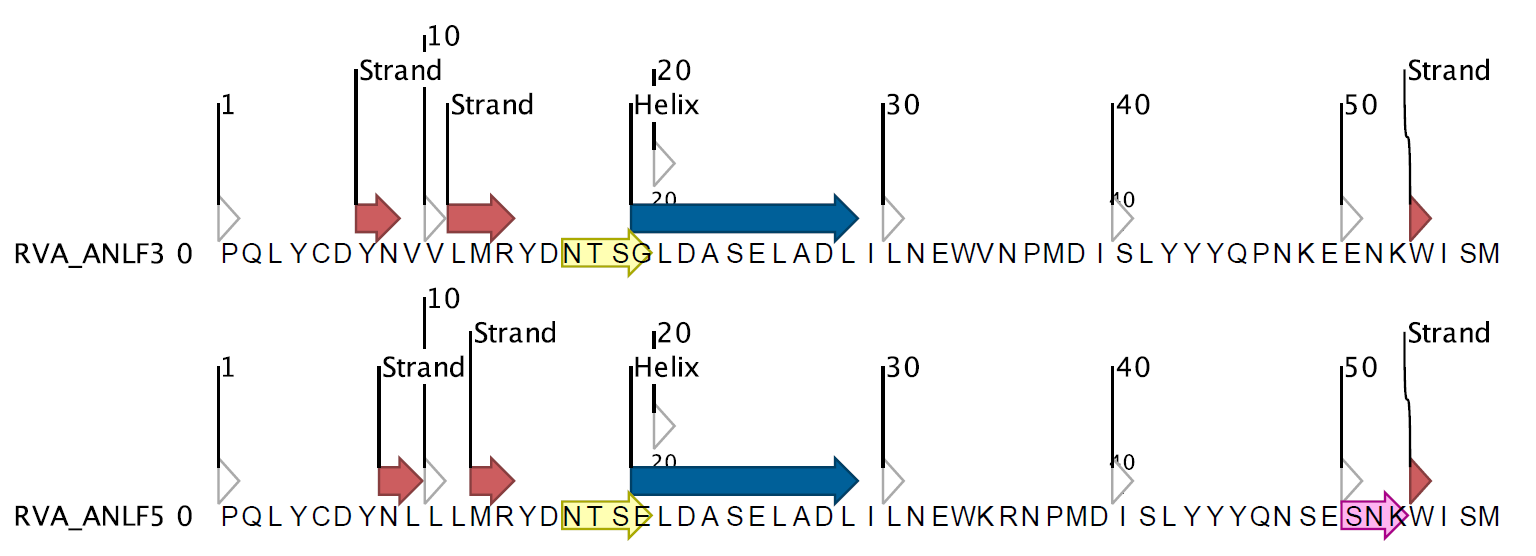
**

**Figure S3. Graphical representation of positions of secondary structures and associated motifs along both sequences.**

The first graph refers to the 2B64I-ANLF3/2018 sequence (abbreviated RVA_ANLF3), whereas the second refers to the 2B64I-ANLF5/2018 sequence (abbreviated RVA_ANLF5). Red arrows denote the start and end positions of the β-sheet, and blue arrows indicate the start and end positions of the α-helix. Yellow arrows highlight sequences defining the N-acetyl glucosamine sites, and the pink arrow highlights the protein kinase C phosphorylation site.


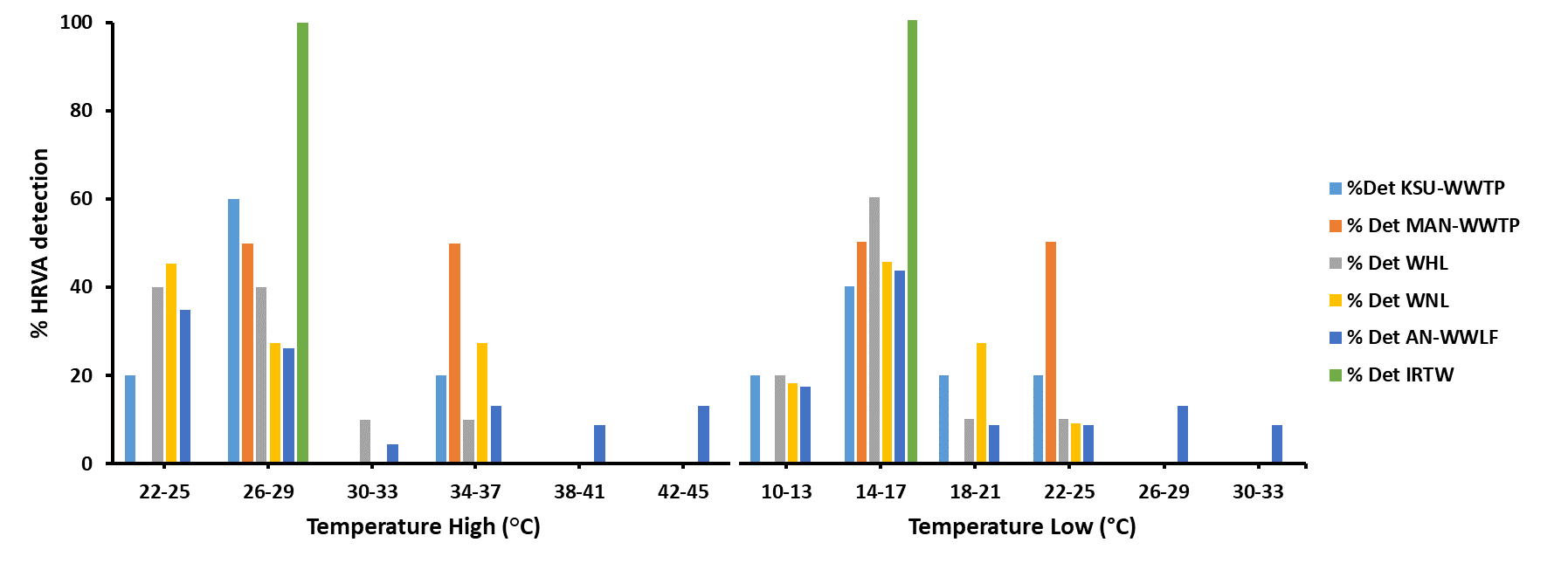
**Figure S4. Impact of temperature variation on the HRVA prevalence in different sampling areas.**

**Table S1. Evolutionary divergence estimates between human rotavirus A sequences**

| **Species 1** | **Species 2** | **Distance (*d*)** |
| --- | --- | --- |
| **RVA/Human-wt/CAN/RT125-07/2008/G2P4** | RVA/TWW/SA/2B64I-KSU1/2018 | **0.00000000** |
| **RVA/Human/JPN/BLU5-vp7/2018/G2P4** | RVA/TWW/SA/2B64I-KSU2/2018 | **0.00000000** |
| **RVA/Human-wt/CHN/G12021182/2012/G2P4** | RVA/TWW/SA/2B64I-KSU2/2018 | **0.00000000** |
| **RVA/Human-wt/TWN/07-96s-498/2007/G2P4** | RVA/TWW/SA/2B64I-KSU2/2018 | **0.00000000** |
| **RVA/Human-wt/CHN/G17081040/2017/G2P4** | RVA/TWW/SA/2B64I-KSU2/2018 | **0.00000000** |
| **RVA/Human/THA/B4285/2017 G2P8** | RVA/TWW/SA/2B64I-KSU2/2018 | **0.00000000** |
| **RVA/Human-wt/CHN/SH-RV76/2015/G2P4** | RVA/TWW/SA/2B64I-KSU2/2018 | **0.00000000** |
| **RVA/Human/KOR/Seoul1433/2010/G2P4** | RVA/TWW/SA/2B64I-KSU2/2018 | **0.00000000** |
| **RVA/Human/RUS/O1270/2011/G2P8** | RVA/TWW/SA/2B64I-KSU2/2018 | **0.00000000** |
| **RVA/Human/RUS/Nov11-N1936/2011/G2P8** | RVA/TWW/SA/2B64I-KSU2/2018 | **0.00000000** |
| **RVA/Human/RUS/O1157/2011/G2P4** | RVA/TWW/SA/2B64I-KSU2/2018 | **0.00000000** |
| **RVA/Human-wt/BEL/BE34/2006/G2P4** | RVA/TWW/SA/2B64I-KSU2/2018 | **0.00000000** |
| **RVA/Human-wt/JPN/B110056/2011/G2P4** | RVA/TWW/SA/2B64I-KSU2/2018 | **0.00000000** |
| **RVA/Human-wt/AUS/CK20027/2006/G2P4** | RVA/TWW/SA/2B64I-KSU2/2018 | **0.00000000** |
| **RVA/Human-wt/IDN/BL-5210/2006/G2P4** | RVA/TWW/SA/2B64I-KSU2/2018 | **0.00000000** |
| **RVA/Human-wt/PAK-HF56/2010/G2P4** | RVA/TWW/SA/2B64I-KSU2/2018 | **0.00000000** |
| RVA/TWW/SA/2B64I-MANF1/2018 | RVA/TWW/SA/2B64I-MANF2/2018 | **0.00000000** |
| RVA/TWW/SA/2B64I-WHL1/2018 | RVA/TWW/SA/2B64I-WHL2/2018 | **0.00000000** |
| RVA/TWW/SA/2B64I-WHL1/2018 | RVA/TWW/SA/2B64I-WHL3/2018 | **0.00000000** |
| RVA/TWW/SA/2B64I-WHL2/2018 | RVA/TWW/SA/2B64I-WHL3/2018 | **0.00000000** |
| RVA/TWW/SA/2B64I-WHL1/2018 | RVA/TWW/SA/2B64I-WHL4/2018 | **0.00000000** |
| RVA/TWW/SA/2B64I-WHL2/2018 | RVA/TWW/SA/2B64I-WHL4/2018 | **0.00000000** |
| RVA/TWW/SA/2B64I-WHL3/2018 | RVA/TWW/SA/2B64I-WHL4/2018 | **0.00000000** |
| RVA/TWW/SA/2B64I-WHL5/2018 | RVA/TWW/SA/2B64I-WHL1/2019 | **0.00000000** |
| RVA/TWW/SA/2B64I-WHL1/2018 | RVA/TWW/SA/2B64I-WHL2/2019 | **0.00000000** |
| RVA/TWW/SA/2B64I-WHL2/2018 | RVA/TWW/SA/2B64I-WHL2/2019 | **0.00000000** |
| RVA/TWW/SA/2B64I-WHL3/2018 | RVA/TWW/SA/2B64I-WHL2/2019 | **0.00000000** |
| RVA/TWW/SA/2B64I-WHL4/2018 | RVA/TWW/SA/2B64I-WHL2/2019 | **0.00000000** |
| RVA/TWW/SA/2B64I-WHL5/2018 | RVA/TWW/SA/2B64I-WNL1/2018 | **0.00000000** |
| RVA/TWW/SA/2B64I-WHL1/2019 | RVA/TWW/SA/2B64I-WNL1/2018 | **0.00000000** |
| RVA/TWW/SA/2B64I-WHL5/2018 | RVA/TWW/SA/2B64I-WNL2/2018 | **0.00000000** |
| RVA/TWW/SA/2B64I-WHL1/2019 | RVA/TWW/SA/2B64I-WNL2/2018 | **0.00000000** |
| RVA/TWW/SA/2B64I-WNL1/2018 | RVA/TWW/SA/2B64I-WNL2/2018 | **0.00000000** |
| RVA/TWW/SA/2B64I-WHL5/2018 | RVA/TWW/SA/2B64I-WNL3/2018 | **0.00000000** |
| RVA/TWW/SA/2B64I-WHL1/2019 | RVA/TWW/SA/2B64I-WNL3/2018 | **0.00000000** |
| RVA/TWW/SA/2B64I-WNL1/2018 | RVA/TWW/SA/2B64I-WNL3/2018 | **0.00000000** |
| RVA/TWW/SA/2B64I-WNL2/2018 | RVA/TWW/SA/2B64I-WNL3/2018 | **0.00000000** |
| RVA/TWW/SA/2B64I-WHL5/2018 | RVA/TWW/SA/2B64I-WNL4/2018 | **0.00000000** |
| RVA/TWW/SA/2B64I-WHL1/2019 | RVA/TWW/SA/2B64I-WNL4/2018 | **0.00000000** |
| RVA/TWW/SA/2B64I-WNL1/2018 | RVA/TWW/SA/2B64I-WNL4/2018 | **0.00000000** |
| RVA/TWW/SA/2B64I-WNL2/2018 | RVA/TWW/SA/2B64I-WNL4/2018 | **0.00000000** |
| RVA/TWW/SA/2B64I-WNL3/2018 | RVA/TWW/SA/2B64I-WNL4/2018 | **0.00000000** |
| RVA/TWW/SA/2B64I-WHL5/2018 | RVA/TWW/SA/2B64I-WNL5/2018 | **0.00000000** |
| RVA/TWW/SA/2B64I-WHL1/2019 | RVA/TWW/SA/2B64I-WNL5/2018 | **0.00000000** |
| RVA/TWW/SA/2B64I-WNL1/2018 | RVA/TWW/SA/2B64I-WNL5/2018 | **0.00000000** |
| RVA/TWW/SA/2B64I-WNL2/2018 | RVA/TWW/SA/2B64I-WNL5/2018 | **0.00000000** |
| RVA/TWW/SA/2B64I-WNL3/2018 | RVA/TWW/SA/2B64I-WNL5/2018 | **0.00000000** |
| RVA/TWW/SA/2B64I-WNL4/2018 | RVA/TWW/SA/2B64I-WNL5/2018 | **0.00000000** |
| RVA/TWW/SA/2B64I-WHL5/2018 | RVA/TWW/SA/2B64I-WNL6/2018 | **0.00000000** |
| RVA/TWW/SA/2B64I-WHL1/2019 | RVA/TWW/SA/2B64I-WNL6/2018 | **0.00000000** |
| RVA/TWW/SA/2B64I-WNL1/2018 | RVA/TWW/SA/2B64I-WNL6/2018 | **0.00000000** |
| RVA/TWW/SA/2B64I-WNL2/2018 | RVA/TWW/SA/2B64I-WNL6/2018 | **0.00000000** |
| RVA/TWW/SA/2B64I-WNL3/2018 | RVA/TWW/SA/2B64I-WNL6/2018 | **0.00000000** |
| RVA/TWW/SA/2B64I-WNL4/2018 | RVA/TWW/SA/2B64I-WNL6/2018 | **0.00000000** |
| RVA/TWW/SA/2B64I-WNL5/2018 | RVA/TWW/SA/2B64I-WNL6/2018 | **0.00000000** |
| RVA/TWW/SA/2B64I-WHL5/2018 | RVA/TWW/SA/2B64I-WNL1/2019 | **0.00000000** |
| RVA/TWW/SA/2B64I-WHL1/2019 | RVA/TWW/SA/2B64I-WNL1/2019 | **0.00000000** |
| RVA/TWW/SA/2B64I-WNL1/2018 | RVA/TWW/SA/2B64I-WNL1/2019 | **0.00000000** |
| RVA/TWW/SA/2B64I-WNL2/2018 | RVA/TWW/SA/2B64I-WNL1/2019 | **0.00000000** |
| RVA/TWW/SA/2B64I-WNL3/2018 | RVA/TWW/SA/2B64I-WNL1/2019 | **0.00000000** |
| RVA/TWW/SA/2B64I-WNL4/2018 | RVA/TWW/SA/2B64I-WNL1/2019 | **0.00000000** |
| RVA/TWW/SA/2B64I-WNL5/2018 | RVA/TWW/SA/2B64I-WNL1/2019 | **0.00000000** |
| RVA/TWW/SA/2B64I-WNL6/2018 | RVA/TWW/SA/2B64I-WNL1/2019 | **0.00000000** |
| RVA/SW/SA/2B64I-ANLF1/2018 | RVA/SW/SA/2B64I-ANLF2/2018 | **0.00000000** |
| RVA/SW/SA/2B64I-ANLF1/2018 | RVA/SW/SA/2B64I-ANLF4/2018 | **0.00000000** |
| RVA/SW/SA/2B64I-ANLF2/2018 | RVA/SW/SA/2B64I-ANLF4/2018 | **0.00000000** |
| RVA/TWW/SA/2B64I-WHL5/2018 | RVA/SW/SA/2B64I-ANLF1/2019 | **0.00000000** |
| RVA/TWW/SA/2B64I-WHL1/2019 | RVA/SW/SA/2B64I-ANLF1/2019 | **0.00000000** |
| RVA/TWW/SA/2B64I-WNL1/2018 | RVA/SW/SA/2B64I-ANLF1/2019 | **0.00000000** |
| RVA/TWW/SA/2B64I-WNL2/2018 | RVA/SW/SA/2B64I-ANLF1/2019 | **0.00000000** |
| RVA/TWW/SA/2B64I-WNL3/2018 | RVA/SW/SA/2B64I-ANLF1/2019 | **0.00000000** |
| RVA/TWW/SA/2B64I-WNL4/2018 | RVA/SW/SA/2B64I-ANLF1/2019 | **0.00000000** |
| RVA/TWW/SA/2B64I-WNL5/2018 | RVA/SW/SA/2B64I-ANLF1/2019 | **0.00000000** |
| RVA/TWW/SA/2B64I-WNL6/2018 | RVA/SW/SA/2B64I-ANLF1/2019 | **0.00000000** |
| RVA/TWW/SA/2B64I-WNL1/2019 | RVA/SW/SA/2B64I-ANLF1/2019 | **0.00000000** |
| RVA/TWW/SA/2B64I-WHL5/2018 | RVA/SW/SA/2B64I-ANLF2/2019 | **0.00000000** |
| RVA/TWW/SA/2B64I-WHL1/2019 | RVA/SW/SA/2B64I-ANLF2/2019 | **0.00000000** |
| RVA/TWW/SA/2B64I-WNL1/2018 | RVA/SW/SA/2B64I-ANLF2/2019 | **0.00000000** |
| RVA/TWW/SA/2B64I-WNL2/2018 | RVA/SW/SA/2B64I-ANLF2/2019 | **0.00000000** |
| RVA/TWW/SA/2B64I-WNL3/2018 | RVA/SW/SA/2B64I-ANLF2/2019 | **0.00000000** |
| RVA/TWW/SA/2B64I-WNL4/2018 | RVA/SW/SA/2B64I-ANLF2/2019 | **0.00000000** |
| RVA/TWW/SA/2B64I-WNL5/2018 | RVA/SW/SA/2B64I-ANLF2/2019 | **0.00000000** |
| RVA/TWW/SA/2B64I-WNL6/2018 | RVA/SW/SA/2B64I-ANLF2/2019 | **0.00000000** |
| RVA/TWW/SA/2B64I-WNL1/2019 | RVA/SW/SA/2B64I-ANLF2/2019 | **0.00000000** |
| RVA/SW/SA/2B64I-ANLF1/2019 | RVA/SW/SA/2B64I-ANLF2/2019 | **0.00000000** |
| RVA/TWW/SA/2B64I-KSU2/2018 | RVA/SW/SA/2B64I-ANLF3/2019 | **0.00000000** |
| RVA/TWW/SA/2B64I-WHL5/2018 | RVA/SW/SA/2B64I-ANLF4/2019 | **0.00000000** |
| RVA/TWW/SA/2B64I-WHL1/2019 | RVA/SW/SA/2B64I-ANLF4/2019 | **0.00000000** |
| RVA/TWW/SA/2B64I-WNL1/2018 | RVA/SW/SA/2B64I-ANLF4/2019 | **0.00000000** |
| RVA/TWW/SA/2B64I-WNL2/2018 | RVA/SW/SA/2B64I-ANLF4/2019 | **0.00000000** |
| RVA/TWW/SA/2B64I-WNL3/2018 | RVA/SW/SA/2B64I-ANLF4/2019 | **0.00000000** |
| RVA/TWW/SA/2B64I-WNL4/2018 | RVA/SW/SA/2B64I-ANLF4/2019 | **0.00000000** |
| RVA/TWW/SA/2B64I-WNL5/2018 | RVA/SW/SA/2B64I-ANLF4/2019 | **0.00000000** |
| RVA/TWW/SA/2B64I-WNL6/2018 | RVA/SW/SA/2B64I-ANLF4/2019 | **0.00000000** |
| RVA/TWW/SA/2B64I-WNL1/2019 | RVA/SW/SA/2B64I-ANLF4/2019 | **0.00000000** |
| RVA/SW/SA/2B64I-ANLF1/2019 | RVA/SW/SA/2B64I-ANLF4/2019 | **0.00000000** |
| RVA/SW/SA/2B64I-ANLF2/2019 | RVA/SW/SA/2B64I-ANLF4/2019 | **0.00000000** |
| RVA/TWW/SA/2B64I-KSU1/2019 | RVA/TWW/SA/2B64I-IRTW1/2018 | **0.00000000** |
| RVA/TWW/SA/2B64I-KSU1/2019 | RVA/TWW/SA/2B64I-IRTW1/2019 | **0.00000000** |
| RVA/TWW/SA/2B64I-IRTW1/2018 | RVA/TWW/SA/2B64I-IRTW1/2019 | **0.00000000** |
| **RVA/Human/JPN/BLU5-vp7/2018/G2P4** | RVA/SW/SA/2B64I-ANLF3/2019 | **0.00000000** |
| **RVA/Human-wt/CHN/G12021182/2012/G2P4** | RVA/SW/SA/2B64I-ANLF3/2019 | **0.00000000** |
| **RVA/Human-wt/TWN/07-96s-498/2007/G2P4** | RVA/SW/SA/2B64I-ANLF3/2019 | **0.00000000** |
| **RVA/Human-wt/CHN/G17081040/2017/G2P4** | RVA/SW/SA/2B64I-ANLF3/2019 | **0.00000000** |
| **RVA/Human/THA/B4285/2017 G2P8** | RVA/SW/SA/2B64I-ANLF3/2019 | **0.00000000** |
| **RVA/Human-wt/CHN/SH-RV76/2015/G2P4** | RVA/SW/SA/2B64I-ANLF3/2019 | **0.00000000** |
| **RVA/Human/KOR/Seoul1433/2010/G2P4** | RVA/SW/SA/2B64I-ANLF3/2019 | **0.00000000** |
| **RVA/Human/RUS/O1270/2011/G2P8** | RVA/SW/SA/2B64I-ANLF3/2019 | **0.00000000** |
| **RVA/Human/RUS/Nov11-N1936/2011/G2P8** | RVA/SW/SA/2B64I-ANLF3/2019 | **0.00000000** |
| **RVA/Human/RUS/O1157/2011/G2P4** | RVA/SW/SA/2B64I-ANLF3/2019 | **0.00000000** |
| **RVA/Human-wt/BEL/BE34/2006/G2P4** | RVA/SW/SA/2B64I-ANLF3/2019 | **0.00000000** |
| **RVA/Human-wt/JPN/B110056/2011/G2P4** | RVA/SW/SA/2B64I-ANLF3/2019 | **0.00000000** |
| **RVA/Human-wt/AUS/CK20027/2006/G2P4** | RVA/SW/SA/2B64I-ANLF3/2019 | **0.00000000** |
| **RVA/Human-wt/IDN/BL-5210/2006/G2P4** | RVA/SW/SA/2B64I-ANLF3/2019 | **0.00000000** |
| **RVA/Human-wt/PAK-HF56/2010/G2P4** | RVA/SW/SA/2B64I-ANLF3/2019 | **0.00000000** |
| RVA/Human-wt/COD/KisB523/2009/G2P4 | RVA/TWW/SA/2B64I-MANF1/2018 | 0.00580662 |
| RVA/Human-wt/COD/KisB523/2009/G2P4 | RVA/TWW/SA/2B64I-MANF2/2018 | 0.00580662 |
| RVA/SW/SA/2B64I-ANLF8/2018 | RVA/SW/SA/2B64I-ANLF9/2018 | 0.00580665 |
| RVA/Human/JPN/BLU5-vp7/2018/G2P4 | RVA/TWW/SA/2B64I-KSU1/2018 | 0.00580672 |
| RVA/Human-wt/CHN/G12021182/2012/G2P4 | RVA/TWW/SA/2B64I-KSU1/2018 | 0.00580672 |
| RVA/Human-wt/TWN/07-96s-498/2007/G2P4 | RVA/TWW/SA/2B64I-KSU1/2018 | 0.00580672 |
| RVA/Human-wt/CHN/G17081040/2017/G2P4 | RVA/TWW/SA/2B64I-KSU1/2018 | 0.00580672 |
| RVA/Human/THA/B4285/2017 G2P8 | RVA/TWW/SA/2B64I-KSU1/2018 | 0.00580672 |
| RVA/Human-wt/CHN/SH-RV76/2015/G2P4 | RVA/TWW/SA/2B64I-KSU1/2018 | 0.00580672 |
| RVA/Human/KOR/Seoul1433/2010/G2P4 | RVA/TWW/SA/2B64I-KSU1/2018 | 0.00580672 |
| RVA/Human/RUS/O1270/2011/G2P8 | RVA/TWW/SA/2B64I-KSU1/2018 | 0.00580672 |
| RVA/Human/RUS/Nov11-N1936/2011/G2P8 | RVA/TWW/SA/2B64I-KSU1/2018 | 0.00580672 |
| RVA/Human/RUS/O1157/2011/G2P4 | RVA/TWW/SA/2B64I-KSU1/2018 | 0.00580672 |
| RVA/Human-wt/BEL/BE34/2006/G2P4 | RVA/TWW/SA/2B64I-KSU1/2018 | 0.00580672 |
| RVA/Human-wt/JPN/B110056/2011/G2P4 | RVA/TWW/SA/2B64I-KSU1/2018 | 0.00580672 |
| RVA/Human-wt/AUS/CK20027/2006/G2P4 | RVA/TWW/SA/2B64I-KSU1/2018 | 0.00580672 |
| RVA/Human-wt/IDN/BL-5210/2006/G2P4 | RVA/TWW/SA/2B64I-KSU1/2018 | 0.00580672 |
| RVA/Human-wt/PAK-HF56/2010/G2P4 | RVA/TWW/SA/2B64I-KSU1/2018 | 0.00580672 |
| RVA/Human-wt/IDN/YK-RVY47/2009/G2P4 | RVA/TWW/SA/2B64I-KSU2/2018 | 0.00580672 |
| RVA/Human-wt/CAN/RT125-07/2008/G2P4 | RVA/TWW/SA/2B64I-KSU2/2018 | 0.00580672 |
| RVA/Human/JPN/BLU5-vp7/2018/G2P4 | RVA/TWW/SA/2B64I-KSU1/2019 | 0.00580672 |
| RVA/Human-wt/CHN/G12021182/2012/G2P4 | RVA/TWW/SA/2B64I-KSU1/2019 | 0.00580672 |
| RVA/Human-wt/TWN/07-96s-498/2007/G2P4 | RVA/TWW/SA/2B64I-KSU1/2019 | 0.00580672 |
| RVA/Human-wt/CHN/G17081040/2017/G2P4 | RVA/TWW/SA/2B64I-KSU1/2019 | 0.00580672 |
| RVA/Human/THA/B4285/2017 G2P8 | RVA/TWW/SA/2B64I-KSU1/2019 | 0.00580672 |
| RVA/Human-wt/CHN/SH-RV76/2015/G2P4 | RVA/TWW/SA/2B64I-KSU1/2019 | 0.00580672 |
| RVA/Human/KOR/Seoul1433/2010/G2P4 | RVA/TWW/SA/2B64I-KSU1/2019 | 0.00580672 |
| RVA/Human/RUS/O1270/2011/G2P8 | RVA/TWW/SA/2B64I-KSU1/2019 | 0.00580672 |
| RVA/Human/RUS/Nov11-N1936/2011/G2P8 | RVA/TWW/SA/2B64I-KSU1/2019 | 0.00580672 |
| RVA/Human/RUS/O1157/2011/G2P4 | RVA/TWW/SA/2B64I-KSU1/2019 | 0.00580672 |
| RVA/Human-wt/BEL/BE34/2006/G2P4 | RVA/TWW/SA/2B64I-KSU1/2019 | 0.00580672 |
| RVA/Human-wt/JPN/B110056/2011/G2P4 | RVA/TWW/SA/2B64I-KSU1/2019 | 0.00580672 |
| RVA/Human-wt/AUS/CK20027/2006/G2P4 | RVA/TWW/SA/2B64I-KSU1/2019 | 0.00580672 |
| RVA/Human-wt/IDN/BL-5210/2006/G2P4 | RVA/TWW/SA/2B64I-KSU1/2019 | 0.00580672 |
| RVA/Human-wt/PAK-HF56/2010/G2P4 | RVA/TWW/SA/2B64I-KSU1/2019 | 0.00580672 |
| RVA/TWW/SA/2B64I-KSU1/2018 | RVA/TWW/SA/2B64I-KSU2/2018 | 0.00580672 |
| RVA/TWW/SA/2B64I-KSU2/2018 | RVA/TWW/SA/2B64I-KSU1/2019 | 0.00580672 |
| RVA/TWW/SA/2B64I-KSU1/2018 | RVA/SW/SA/2B64I-ANLF3/2019 | 0.00580672 |
| RVA/TWW/SA/2B64I-KSU1/2019 | RVA/SW/SA/2B64I-ANLF3/2019 | 0.00580672 |
| RVA/TWW/SA/2B64I-KSU2/2018 | RVA/TWW/SA/2B64I-IRTW1/2018 | 0.00580672 |
| RVA/SW/SA/2B64I-ANLF3/2019 | RVA/TWW/SA/2B64I-IRTW1/2018 | 0.00580672 |
| RVA/TWW/SA/2B64I-KSU2/2018 | RVA/TWW/SA/2B64I-IRTW1/2019 | 0.00580672 |
| RVA/SW/SA/2B64I-ANLF3/2019 | RVA/TWW/SA/2B64I-IRTW1/2019 | 0.00580672 |
| RVA/Human-wt/IDN/YK-RVY47/2009/G2P4 | RVA/SW/SA/2B64I-ANLF3/2019 | 0.00580672 |
| RVA/Human-wt/CAN/RT125-07/2008/G2P4 | RVA/SW/SA/2B64I-ANLF3/2019 | 0.00580672 |
| RVA/Human/JPN/BLU5-vp7/2018/G2P4 | RVA/TWW/SA/2B64I-IRTW1/2018 | 0.00580672 |
| RVA/Human-wt/CHN/G12021182/2012/G2P4 | RVA/TWW/SA/2B64I-IRTW1/2018 | 0.00580672 |
| RVA/Human-wt/TWN/07-96s-498/2007/G2P4 | RVA/TWW/SA/2B64I-IRTW1/2018 | 0.00580672 |
| RVA/Human-wt/CHN/G17081040/2017/G2P4 | RVA/TWW/SA/2B64I-IRTW1/2018 | 0.00580672 |
| RVA/Human/THA/B4285/2017 G2P8 | RVA/TWW/SA/2B64I-IRTW1/2018 | 0.00580672 |
| RVA/Human-wt/CHN/SH-RV76/2015/G2P4 | RVA/TWW/SA/2B64I-IRTW1/2018 | 0.00580672 |
| RVA/Human/KOR/Seoul1433/2010/G2P4 | RVA/TWW/SA/2B64I-IRTW1/2018 | 0.00580672 |
| RVA/Human/RUS/O1270/2011/G2P8 | RVA/TWW/SA/2B64I-IRTW1/2018 | 0.00580672 |
| RVA/Human/RUS/Nov11-N1936/2011/G2P8 | RVA/TWW/SA/2B64I-IRTW1/2018 | 0.00580672 |
| RVA/Human/RUS/O1157/2011/G2P4 | RVA/TWW/SA/2B64I-IRTW1/2018 | 0.00580672 |
| RVA/Human-wt/BEL/BE34/2006/G2P4 | RVA/TWW/SA/2B64I-IRTW1/2018 | 0.00580672 |
| RVA/Human-wt/JPN/B110056/2011/G2P4 | RVA/TWW/SA/2B64I-IRTW1/2018 | 0.00580672 |
| RVA/Human-wt/AUS/CK20027/2006/G2P4 | RVA/TWW/SA/2B64I-IRTW1/2018 | 0.00580672 |
| RVA/Human-wt/IDN/BL-5210/2006/G2P4 | RVA/TWW/SA/2B64I-IRTW1/2018 | 0.00580672 |
| RVA/Human-wt/PAK-HF56/2010/G2P4 | RVA/TWW/SA/2B64I-IRTW1/2018 | 0.00580672 |
| RVA/Human/JPN/BLU5-vp7/2018/G2P4 | RVA/TWW/SA/2B64I-IRTW1/2019 | 0.00580672 |
| RVA/Human-wt/CHN/G12021182/2012/G2P4 | RVA/TWW/SA/2B64I-IRTW1/2019 | 0.00580672 |
| RVA/Human-wt/TWN/07-96s-498/2007/G2P4 | RVA/TWW/SA/2B64I-IRTW1/2019 | 0.00580672 |
| RVA/Human-wt/CHN/G17081040/2017/G2P4 | RVA/TWW/SA/2B64I-IRTW1/2019 | 0.00580672 |
| RVA/Human/THA/B4285/2017 G2P8 | RVA/TWW/SA/2B64I-IRTW1/2019 | 0.00580672 |
| RVA/Human-wt/CHN/SH-RV76/2015/G2P4 | RVA/TWW/SA/2B64I-IRTW1/2019 | 0.00580672 |
| RVA/Human/KOR/Seoul1433/2010/G2P4 | RVA/TWW/SA/2B64I-IRTW1/2019 | 0.00580672 |
| RVA/Human/RUS/O1270/2011/G2P8 | RVA/TWW/SA/2B64I-IRTW1/2019 | 0.00580672 |
| RVA/Human/RUS/Nov11-N1936/2011/G2P8 | RVA/TWW/SA/2B64I-IRTW1/2019 | 0.00580672 |
| RVA/Human/RUS/O1157/2011/G2P4 | RVA/TWW/SA/2B64I-IRTW1/2019 | 0.00580672 |
| RVA/Human-wt/BEL/BE34/2006/G2P4 | RVA/TWW/SA/2B64I-IRTW1/2019 | 0.00580672 |
| RVA/Human-wt/JPN/B110056/2011/G2P4 | RVA/TWW/SA/2B64I-IRTW1/2019 | 0.00580672 |
| RVA/Human-wt/AUS/CK20027/2006/G2P4 | RVA/TWW/SA/2B64I-IRTW1/2019 | 0.00580672 |
| RVA/Human-wt/IDN/BL-5210/2006/G2P4 | RVA/TWW/SA/2B64I-IRTW1/2019 | 0.00580672 |
| RVA/Human-wt/PAK-HF56/2010/G2P4 | RVA/TWW/SA/2B64I-IRTW1/2019 | 0.00580672 |
| RVA/SW/SA/2B64I-ANLF5/2018 | RVA/SW/SA/2B64I-ANLF6/2018 | 0.00580695 |
| RVA/Sew/SP/R12A-vp7/2016/G2P8 | RVA/TWW/SA/2B64I-KSU2/2018 | 0.00581903 |
| RVA/Human-wt/THA/SiRAV-667/2017/G2P8 | RVA/TWW/SA/2B64I-KSU2/2018 | 0.00581903 |
| RVA/Human-wt/ETH/BD522/2016/G2P4 | RVA/TWW/SA/2B64I-KSU2/2018 | 0.00581903 |
| RVA/Human-wt/PAK/PAK205/2015/G2P4 | RVA/TWW/SA/2B64I-KSU2/2018 | 0.00581903 |
| RVA/Human-wt/PAK/NIH-BBH-4705/2015/G2P4 | RVA/TWW/SA/2B64I-KSU2/2018 | 0.00581903 |
| RVA/Human-wt/PAK/NIH-KGH-4455/2016/G2P4 | RVA/TWW/SA/2B64I-KSU2/2018 | 0.00581903 |
| RVA/Human-wt/COD/KisB523/2009/G2P4 | RVA/TWW/SA/2B64I-KSU2/2018 | 0.00581903 |
| RVA/Human/THA/B6295/2018/G2P8 | RVA/TWW/SA/2B64I-KSU2/2018 | 0.00581903 |
| RVA/Human/THA/B5613/2018/G2P4 | RVA/TWW/SA/2B64I-KSU2/2018 | 0.00581903 |
| RVA/Human-wt/RUS/NS18-A1455/2018/G2P4 | RVA/TWW/SA/2B64I-KSU2/2018 | 0.00581903 |
| RVA/Human/THA/B5581/2018/G2P8 | RVA/TWW/SA/2B64I-KSU2/2018 | 0.00581903 |
| RVA/Sew/SP/R12A-vp7/2016/G2P8 | RVA/SW/SA/2B64I-ANLF3/2019 | 0.00581903 |
| RVA/Human-wt/THA/SiRAV-667/2017/G2P8 | RVA/SW/SA/2B64I-ANLF3/2019 | 0.00581903 |
| RVA/Human-wt/ETH/BD522/2016/G2P4 | RVA/SW/SA/2B64I-ANLF3/2019 | 0.00581903 |
| RVA/Human-wt/PAK/PAK205/2015/G2P4 | RVA/SW/SA/2B64I-ANLF3/2019 | 0.00581903 |
| RVA/Human-wt/PAK/NIH-BBH-4705/2015/G2P4 | RVA/SW/SA/2B64I-ANLF3/2019 | 0.00581903 |
| RVA/Human-wt/PAK/NIH-KGH-4455/2016/G2P4 | RVA/SW/SA/2B64I-ANLF3/2019 | 0.00581903 |
| RVA/Human-wt/COD/KisB523/2009/G2P4 | RVA/SW/SA/2B64I-ANLF3/2019 | 0.00581903 |
| RVA/Human/THA/B6295/2018/G2P8 | RVA/SW/SA/2B64I-ANLF3/2019 | 0.00581903 |
| RVA/Human/THA/B5613/2018/G2P4 | RVA/SW/SA/2B64I-ANLF3/2019 | 0.00581903 |
| RVA/Human-wt/RUS/NS18-A1455/2018/G2P4 | RVA/SW/SA/2B64I-ANLF3/2019 | 0.00581903 |
| RVA/Human/THA/B5581/2018/G2P8 | RVA/SW/SA/2B64I-ANLF3/2019 | 0.00581903 |
| RVA/Human-wt/SGP/NV-16-161/2016/G2P4 | RVA/TWW/SA/2B64I-KSU2/2018 | 0.00581941 |
| RVA/Human/RUS/S12-14/2012/G2P4 | RVA/TWW/SA/2B64I-KSU2/2018 | 0.00581941 |
| RVA/Human/JPN/BLU5-vp7/2018/G2P4 | RVA/TWW/SA/2B64I-KSU3/2018 | 0.00581941 |
| RVA/Human-wt/CHN/G12021182/2012/G2P4 | RVA/TWW/SA/2B64I-KSU3/2018 | 0.00581941 |
| RVA/Human-wt/TWN/07-96s-498/2007/G2P4 | RVA/TWW/SA/2B64I-KSU3/2018 | 0.00581941 |
| RVA/Human-wt/CHN/G17081040/2017/G2P4 | RVA/TWW/SA/2B64I-KSU3/2018 | 0.00581941 |
| RVA/Human/THA/B4285/2017 G2P8 | RVA/TWW/SA/2B64I-KSU3/2018 | 0.00581941 |
| RVA/Human-wt/CHN/SH-RV76/2015/G2P4 | RVA/TWW/SA/2B64I-KSU3/2018 | 0.00581941 |
| RVA/Human/KOR/Seoul1433/2010/G2P4 | RVA/TWW/SA/2B64I-KSU3/2018 | 0.00581941 |
| RVA/Human/RUS/O1270/2011/G2P8 | RVA/TWW/SA/2B64I-KSU3/2018 | 0.00581941 |
| RVA/Human/RUS/Nov11-N1936/2011/G2P8 | RVA/TWW/SA/2B64I-KSU3/2018 | 0.00581941 |
| RVA/Human/RUS/O1157/2011/G2P4 | RVA/TWW/SA/2B64I-KSU3/2018 | 0.00581941 |
| RVA/Human-wt/BEL/BE34/2006/G2P4 | RVA/TWW/SA/2B64I-KSU3/2018 | 0.00581941 |
| RVA/Human-wt/JPN/B110056/2011/G2P4 | RVA/TWW/SA/2B64I-KSU3/2018 | 0.00581941 |
| RVA/Human-wt/AUS/CK20027/2006/G2P4 | RVA/TWW/SA/2B64I-KSU3/2018 | 0.00581941 |
| RVA/Human-wt/IDN/BL-5210/2006/G2P4 | RVA/TWW/SA/2B64I-KSU3/2018 | 0.00581941 |
| RVA/Human-wt/PAK-HF56/2010/G2P4 | RVA/TWW/SA/2B64I-KSU3/2018 | 0.00581941 |
| RVA/TWW/SA/2B64I-KSU2/2018 | RVA/TWW/SA/2B64I-KSU3/2018 | 0.00581941 |
| RVA/TWW/SA/2B64I-WHL5/2018 | RVA/SW/SA/2B64I-ANLF8/2018 | 0.00581941 |
| RVA/TWW/SA/2B64I-WHL1/2019 | RVA/SW/SA/2B64I-ANLF8/2018 | 0.00581941 |
| RVA/TWW/SA/2B64I-WNL1/2018 | RVA/SW/SA/2B64I-ANLF8/2018 | 0.00581941 |
| RVA/TWW/SA/2B64I-WNL2/2018 | RVA/SW/SA/2B64I-ANLF8/2018 | 0.00581941 |
| RVA/TWW/SA/2B64I-WNL3/2018 | RVA/SW/SA/2B64I-ANLF8/2018 | 0.00581941 |
| RVA/TWW/SA/2B64I-WNL4/2018 | RVA/SW/SA/2B64I-ANLF8/2018 | 0.00581941 |
| RVA/TWW/SA/2B64I-WNL5/2018 | RVA/SW/SA/2B64I-ANLF8/2018 | 0.00581941 |
| RVA/TWW/SA/2B64I-WNL6/2018 | RVA/SW/SA/2B64I-ANLF8/2018 | 0.00581941 |
| RVA/TWW/SA/2B64I-WNL1/2019 | RVA/SW/SA/2B64I-ANLF8/2018 | 0.00581941 |
| RVA/SW/SA/2B64I-ANLF8/2018 | RVA/SW/SA/2B64I-ANLF1/2019 | 0.00581941 |
| RVA/SW/SA/2B64I-ANLF8/2018 | RVA/SW/SA/2B64I-ANLF2/2019 | 0.00581941 |
| RVA/TWW/SA/2B64I-KSU3/2018 | RVA/SW/SA/2B64I-ANLF3/2019 | 0.00581941 |
| RVA/SW/SA/2B64I-ANLF8/2018 | RVA/SW/SA/2B64I-ANLF4/2019 | 0.00581941 |
| RVA/Human-wt/SGP/NV-16-161/2016/G2P4 | RVA/SW/SA/2B64I-ANLF3/2019 | 0.00581941 |
| RVA/Human/RUS/S12-14/2012/G2P4 | RVA/SW/SA/2B64I-ANLF3/2019 | 0.00581941 |
| RVA/Human/JPN/BLU5-vp7/2018/G2P4 | RVA/TWW/SA/2B64I-MANF1/2018 | 0.01165974 |
| RVA/Human-wt/CHN/G12021182/2012/G2P4 | RVA/TWW/SA/2B64I-MANF1/2018 | 0.01165974 |
| RVA/Human-wt/TWN/07-96s-498/2007/G2P4 | RVA/TWW/SA/2B64I-MANF1/2018 | 0.01165974 |
| RVA/Human-wt/CHN/G17081040/2017/G2P4 | RVA/TWW/SA/2B64I-MANF1/2018 | 0.01165974 |
| RVA/Human/THA/B4285/2017 G2P8 | RVA/TWW/SA/2B64I-MANF1/2018 | 0.01165974 |
| RVA/Human-wt/CHN/SH-RV76/2015/G2P4 | RVA/TWW/SA/2B64I-MANF1/2018 | 0.01165974 |
| RVA/Human/KOR/Seoul1433/2010/G2P4 | RVA/TWW/SA/2B64I-MANF1/2018 | 0.01165974 |
| RVA/Human/RUS/O1270/2011/G2P8 | RVA/TWW/SA/2B64I-MANF1/2018 | 0.01165974 |
| RVA/Human/RUS/Nov11-N1936/2011/G2P8 | RVA/TWW/SA/2B64I-MANF1/2018 | 0.01165974 |
| RVA/Human/RUS/O1157/2011/G2P4 | RVA/TWW/SA/2B64I-MANF1/2018 | 0.01165974 |
| RVA/Human-wt/BEL/BE34/2006/G2P4 | RVA/TWW/SA/2B64I-MANF1/2018 | 0.01165974 |
| RVA/Human-wt/JPN/B110056/2011/G2P4 | RVA/TWW/SA/2B64I-MANF1/2018 | 0.01165974 |
| RVA/Human-wt/AUS/CK20027/2006/G2P4 | RVA/TWW/SA/2B64I-MANF1/2018 | 0.01165974 |
| RVA/Human-wt/IDN/BL-5210/2006/G2P4 | RVA/TWW/SA/2B64I-MANF1/2018 | 0.01165974 |
| RVA/Human-wt/PAK-HF56/2010/G2P4 | RVA/TWW/SA/2B64I-MANF1/2018 | 0.01165974 |
| RVA/Human/JPN/BLU5-vp7/2018/G2P4 | RVA/TWW/SA/2B64I-MANF2/2018 | 0.01165974 |
| RVA/Human-wt/CHN/G12021182/2012/G2P4 | RVA/TWW/SA/2B64I-MANF2/2018 | 0.01165974 |
| RVA/Human-wt/TWN/07-96s-498/2007/G2P4 | RVA/TWW/SA/2B64I-MANF2/2018 | 0.01165974 |
| RVA/Human-wt/CHN/G17081040/2017/G2P4 | RVA/TWW/SA/2B64I-MANF2/2018 | 0.01165974 |
| RVA/Human/THA/B4285/2017 G2P8 | RVA/TWW/SA/2B64I-MANF2/2018 | 0.01165974 |
| RVA/Human-wt/CHN/SH-RV76/2015/G2P4 | RVA/TWW/SA/2B64I-MANF2/2018 | 0.01165974 |
| RVA/Human/KOR/Seoul1433/2010/G2P4 | RVA/TWW/SA/2B64I-MANF2/2018 | 0.01165974 |
| RVA/Human/RUS/O1270/2011/G2P8 | RVA/TWW/SA/2B64I-MANF2/2018 | 0.01165974 |
| RVA/Human/RUS/Nov11-N1936/2011/G2P8 | RVA/TWW/SA/2B64I-MANF2/2018 | 0.01165974 |
| RVA/Human/RUS/O1157/2011/G2P4 | RVA/TWW/SA/2B64I-MANF2/2018 | 0.01165974 |
| RVA/Human-wt/BEL/BE34/2006/G2P4 | RVA/TWW/SA/2B64I-MANF2/2018 | 0.01165974 |
| RVA/Human-wt/JPN/B110056/2011/G2P4 | RVA/TWW/SA/2B64I-MANF2/2018 | 0.01165974 |
| RVA/Human-wt/AUS/CK20027/2006/G2P4 | RVA/TWW/SA/2B64I-MANF2/2018 | 0.01165974 |
| RVA/Human-wt/IDN/BL-5210/2006/G2P4 | RVA/TWW/SA/2B64I-MANF2/2018 | 0.01165974 |
| RVA/Human-wt/PAK-HF56/2010/G2P4 | RVA/TWW/SA/2B64I-MANF2/2018 | 0.01165974 |
| RVA/TWW/SA/2B64I-KSU2/2018 | RVA/TWW/SA/2B64I-MANF1/2018 | 0.01165974 |
| RVA/TWW/SA/2B64I-KSU2/2018 | RVA/TWW/SA/2B64I-MANF2/2018 | 0.01165974 |
| RVA/TWW/SA/2B64I-MANF1/2018 | RVA/SW/SA/2B64I-ANLF3/2019 | 0.01165974 |
| RVA/TWW/SA/2B64I-MANF2/2018 | RVA/SW/SA/2B64I-ANLF3/2019 | 0.01165974 |
| RVA/Sew/SP/R12A-vp7/2016/G2P8 | RVA/TWW/SA/2B64I-KSU1/2018 | 0.01165996 |
| RVA/Human-wt/THA/SiRAV-667/2017/G2P8 | RVA/TWW/SA/2B64I-KSU1/2018 | 0.01165996 |
| RVA/Human-wt/ETH/BD522/2016/G2P4 | RVA/TWW/SA/2B64I-KSU1/2018 | 0.01165996 |
| RVA/Human-wt/PAK/PAK205/2015/G2P4 | RVA/TWW/SA/2B64I-KSU1/2018 | 0.01165996 |
| RVA/Human-wt/PAK/NIH-BBH-4705/2015/G2P4 | RVA/TWW/SA/2B64I-KSU1/2018 | 0.01165996 |
| RVA/Human-wt/PAK/NIH-KGH-4455/2016/G2P4 | RVA/TWW/SA/2B64I-KSU1/2018 | 0.01165996 |
| RVA/Human-wt/COD/KisB523/2009/G2P4 | RVA/TWW/SA/2B64I-KSU1/2018 | 0.01165996 |
| RVA/Human/THA/B6295/2018/G2P8 | RVA/TWW/SA/2B64I-KSU1/2018 | 0.01165996 |
| RVA/Human/THA/B5613/2018/G2P4 | RVA/TWW/SA/2B64I-KSU1/2018 | 0.01165996 |
| RVA/Human-wt/RUS/NS18-A1455/2018/G2P4 | RVA/TWW/SA/2B64I-KSU1/2018 | 0.01165996 |
| RVA/Human/THA/B5581/2018/G2P8 | RVA/TWW/SA/2B64I-KSU1/2018 | 0.01165996 |
| RVA/Sew/SP/R12A-vp7/2016/G2P8 | RVA/TWW/SA/2B64I-KSU1/2019 | 0.01165996 |
| RVA/Human-wt/THA/SiRAV-667/2017/G2P8 | RVA/TWW/SA/2B64I-KSU1/2019 | 0.01165996 |
| RVA/Human-wt/ETH/BD522/2016/G2P4 | RVA/TWW/SA/2B64I-KSU1/2019 | 0.01165996 |
| RVA/Human-wt/PAK/PAK205/2015/G2P4 | RVA/TWW/SA/2B64I-KSU1/2019 | 0.01165996 |
| RVA/Human-wt/PAK/NIH-BBH-4705/2015/G2P4 | RVA/TWW/SA/2B64I-KSU1/2019 | 0.01165996 |
| RVA/Human-wt/PAK/NIH-KGH-4455/2016/G2P4 | RVA/TWW/SA/2B64I-KSU1/2019 | 0.01165996 |
| RVA/Human-wt/COD/KisB523/2009/G2P4 | RVA/TWW/SA/2B64I-KSU1/2019 | 0.01165996 |
| RVA/Human/THA/B6295/2018/G2P8 | RVA/TWW/SA/2B64I-KSU1/2019 | 0.01165996 |
| RVA/Human/THA/B5613/2018/G2P4 | RVA/TWW/SA/2B64I-KSU1/2019 | 0.01165996 |
| RVA/Human-wt/RUS/NS18-A1455/2018/G2P4 | RVA/TWW/SA/2B64I-KSU1/2019 | 0.01165996 |
| RVA/Human/THA/B5581/2018/G2P8 | RVA/TWW/SA/2B64I-KSU1/2019 | 0.01165996 |
| RVA/TWW/SA/2B64I-WHL5/2018 | RVA/SW/SA/2B64I-ANLF9/2018 | 0.01165996 |
| RVA/TWW/SA/2B64I-WHL1/2019 | RVA/SW/SA/2B64I-ANLF9/2018 | 0.01165996 |
| RVA/TWW/SA/2B64I-WNL1/2018 | RVA/SW/SA/2B64I-ANLF9/2018 | 0.01165996 |
| RVA/TWW/SA/2B64I-WNL2/2018 | RVA/SW/SA/2B64I-ANLF9/2018 | 0.01165996 |
| RVA/TWW/SA/2B64I-WNL3/2018 | RVA/SW/SA/2B64I-ANLF9/2018 | 0.01165996 |
| RVA/TWW/SA/2B64I-WNL4/2018 | RVA/SW/SA/2B64I-ANLF9/2018 | 0.01165996 |
| RVA/TWW/SA/2B64I-WNL5/2018 | RVA/SW/SA/2B64I-ANLF9/2018 | 0.01165996 |
| RVA/TWW/SA/2B64I-WNL6/2018 | RVA/SW/SA/2B64I-ANLF9/2018 | 0.01165996 |
| RVA/TWW/SA/2B64I-WNL1/2019 | RVA/SW/SA/2B64I-ANLF9/2018 | 0.01165996 |
| RVA/SW/SA/2B64I-ANLF9/2018 | RVA/SW/SA/2B64I-ANLF1/2019 | 0.01165996 |
| RVA/SW/SA/2B64I-ANLF9/2018 | RVA/SW/SA/2B64I-ANLF2/2019 | 0.01165996 |
| RVA/SW/SA/2B64I-ANLF9/2018 | RVA/SW/SA/2B64I-ANLF4/2019 | 0.01165996 |
| RVA/Sew/SP/R12A-vp7/2016/G2P8 | RVA/TWW/SA/2B64I-IRTW1/2018 | 0.01165996 |
| RVA/Human-wt/THA/SiRAV-667/2017/G2P8 | RVA/TWW/SA/2B64I-IRTW1/2018 | 0.01165996 |
| RVA/Human-wt/ETH/BD522/2016/G2P4 | RVA/TWW/SA/2B64I-IRTW1/2018 | 0.01165996 |
| RVA/Human-wt/PAK/PAK205/2015/G2P4 | RVA/TWW/SA/2B64I-IRTW1/2018 | 0.01165996 |
| RVA/Human-wt/PAK/NIH-BBH-4705/2015/G2P4 | RVA/TWW/SA/2B64I-IRTW1/2018 | 0.01165996 |
| RVA/Human-wt/PAK/NIH-KGH-4455/2016/G2P4 | RVA/TWW/SA/2B64I-IRTW1/2018 | 0.01165996 |
| RVA/Human-wt/COD/KisB523/2009/G2P4 | RVA/TWW/SA/2B64I-IRTW1/2018 | 0.01165996 |
| RVA/Human/THA/B6295/2018/G2P8 | RVA/TWW/SA/2B64I-IRTW1/2018 | 0.01165996 |
| RVA/Human/THA/B5613/2018/G2P4 | RVA/TWW/SA/2B64I-IRTW1/2018 | 0.01165996 |
| RVA/Human-wt/RUS/NS18-A1455/2018/G2P4 | RVA/TWW/SA/2B64I-IRTW1/2018 | 0.01165996 |
| RVA/Human/THA/B5581/2018/G2P8 | RVA/TWW/SA/2B64I-IRTW1/2018 | 0.01165996 |
| RVA/Sew/SP/R12A-vp7/2016/G2P8 | RVA/TWW/SA/2B64I-IRTW1/2019 | 0.01165996 |
| RVA/Human-wt/THA/SiRAV-667/2017/G2P8 | RVA/TWW/SA/2B64I-IRTW1/2019 | 0.01165996 |
| RVA/Human-wt/ETH/BD522/2016/G2P4 | RVA/TWW/SA/2B64I-IRTW1/2019 | 0.01165996 |
| RVA/Human-wt/PAK/PAK205/2015/G2P4 | RVA/TWW/SA/2B64I-IRTW1/2019 | 0.01165996 |
| RVA/Human-wt/PAK/NIH-BBH-4705/2015/G2P4 | RVA/TWW/SA/2B64I-IRTW1/2019 | 0.01165996 |
| RVA/Human-wt/PAK/NIH-KGH-4455/2016/G2P4 | RVA/TWW/SA/2B64I-IRTW1/2019 | 0.01165996 |
| RVA/Human-wt/COD/KisB523/2009/G2P4 | RVA/TWW/SA/2B64I-IRTW1/2019 | 0.01165996 |
| RVA/Human/THA/B6295/2018/G2P8 | RVA/TWW/SA/2B64I-IRTW1/2019 | 0.01165996 |
| RVA/Human/THA/B5613/2018/G2P4 | RVA/TWW/SA/2B64I-IRTW1/2019 | 0.01165996 |
| RVA/Human-wt/RUS/NS18-A1455/2018/G2P4 | RVA/TWW/SA/2B64I-IRTW1/2019 | 0.01165996 |
| RVA/Human/THA/B5581/2018/G2P8 | RVA/TWW/SA/2B64I-IRTW1/2019 | 0.01165996 |
| RVA/Human-wt/SGP/NV-16-161/2016/G2P4 | RVA/TWW/SA/2B64I-KSU1/2018 | 0.01166043 |
| RVA/Human/RUS/S12-14/2012/G2P4 | RVA/TWW/SA/2B64I-KSU1/2018 | 0.01166043 |
| RVA/Human-wt/IDN/YK-RVY47/2009/G2P4 | RVA/TWW/SA/2B64I-KSU3/2018 | 0.01166043 |
| RVA/Human-wt/CAN/RT125-07/2008/G2P4 | RVA/TWW/SA/2B64I-KSU3/2018 | 0.01166043 |
| RVA/Human-wt/SGP/NV-16-161/2016/G2P4 | RVA/TWW/SA/2B64I-KSU1/2019 | 0.01166043 |
| RVA/Human/RUS/S12-14/2012/G2P4 | RVA/TWW/SA/2B64I-KSU1/2019 | 0.01166043 |
| RVA/TWW/SA/2B64I-KSU1/2018 | RVA/TWW/SA/2B64I-KSU3/2018 | 0.01166043 |
| RVA/TWW/SA/2B64I-KSU3/2018 | RVA/TWW/SA/2B64I-KSU1/2019 | 0.01166043 |
| RVA/TWW/SA/2B64I-KSU3/2018 | RVA/TWW/SA/2B64I-IRTW1/2018 | 0.01166043 |
| RVA/TWW/SA/2B64I-KSU3/2018 | RVA/TWW/SA/2B64I-IRTW1/2019 | 0.01166043 |
| RVA/Human-wt/SGP/NV-16-161/2016/G2P4 | RVA/TWW/SA/2B64I-IRTW1/2018 | 0.01166043 |
| RVA/Human/RUS/S12-14/2012/G2P4 | RVA/TWW/SA/2B64I-IRTW1/2018 | 0.01166043 |
| RVA/Human-wt/SGP/NV-16-161/2016/G2P4 | RVA/TWW/SA/2B64I-IRTW1/2019 | 0.01166043 |
| RVA/Human/RUS/S12-14/2012/G2P4 | RVA/TWW/SA/2B64I-IRTW1/2019 | 0.01166043 |
| RVA/Human-wt/IDN/YK-RVY47/2009/G2P4 | RVA/TWW/SA/2B64I-KSU1/2018 | 0.01166707 |
| RVA/Human-wt/IDN/YK-RVY47/2009/G2P4 | RVA/TWW/SA/2B64I-KSU1/2019 | 0.01166707 |
| RVA/Human-wt/CAN/RT125-07/2008/G2P4 | RVA/TWW/SA/2B64I-KSU1/2019 | 0.01166707 |
| RVA/TWW/SA/2B64I-KSU1/2018 | RVA/TWW/SA/2B64I-KSU1/2019 | 0.01166707 |
| RVA/TWW/SA/2B64I-WHL5/2018 | RVA/SW/SA/2B64I-ANLF7/2018 | 0.01166707 |
| RVA/TWW/SA/2B64I-WHL1/2019 | RVA/SW/SA/2B64I-ANLF7/2018 | 0.01166707 |
| RVA/TWW/SA/2B64I-WNL1/2018 | RVA/SW/SA/2B64I-ANLF7/2018 | 0.01166707 |
| RVA/TWW/SA/2B64I-WNL2/2018 | RVA/SW/SA/2B64I-ANLF7/2018 | 0.01166707 |
| RVA/TWW/SA/2B64I-WNL3/2018 | RVA/SW/SA/2B64I-ANLF7/2018 | 0.01166707 |
| RVA/TWW/SA/2B64I-WNL4/2018 | RVA/SW/SA/2B64I-ANLF7/2018 | 0.01166707 |
| RVA/TWW/SA/2B64I-WNL5/2018 | RVA/SW/SA/2B64I-ANLF7/2018 | 0.01166707 |
| RVA/TWW/SA/2B64I-WNL6/2018 | RVA/SW/SA/2B64I-ANLF7/2018 | 0.01166707 |
| RVA/TWW/SA/2B64I-WNL1/2019 | RVA/SW/SA/2B64I-ANLF7/2018 | 0.01166707 |
| RVA/SW/SA/2B64I-ANLF7/2018 | RVA/SW/SA/2B64I-ANLF1/2019 | 0.01166707 |
| RVA/SW/SA/2B64I-ANLF7/2018 | RVA/SW/SA/2B64I-ANLF2/2019 | 0.01166707 |
| RVA/SW/SA/2B64I-ANLF7/2018 | RVA/SW/SA/2B64I-ANLF4/2019 | 0.01166707 |
| RVA/TWW/SA/2B64I-KSU1/2018 | RVA/TWW/SA/2B64I-IRTW1/2018 | 0.01166707 |
| RVA/TWW/SA/2B64I-KSU1/2018 | RVA/TWW/SA/2B64I-IRTW1/2019 | 0.01166707 |
| RVA/Human-wt/IDN/YK-RVY47/2009/G2P4 | RVA/TWW/SA/2B64I-IRTW1/2018 | 0.01166707 |
| RVA/Human-wt/CAN/RT125-07/2008/G2P4 | RVA/TWW/SA/2B64I-IRTW1/2018 | 0.01166707 |
| RVA/Human-wt/IDN/YK-RVY47/2009/G2P4 | RVA/TWW/SA/2B64I-IRTW1/2019 | 0.01166707 |
| RVA/Human-wt/CAN/RT125-07/2008/G2P4 | RVA/TWW/SA/2B64I-IRTW1/2019 | 0.01166707 |
| RVA/SW/SA/2B64I-ANLF1/2018 | RVA/SW/SA/2B64I-ANLF3/2018 | 0.01166722 |
| RVA/SW/SA/2B64I-ANLF2/2018 | RVA/SW/SA/2B64I-ANLF3/2018 | 0.01166722 |
| RVA/SW/SA/2B64I-ANLF3/2018 | RVA/SW/SA/2B64I-ANLF4/2018 | 0.01166722 |

The table comprised only sequences having a divergence distance (*d*) of up to 0.01167 to our HRVA sequences. Null distances were displayed in red. Moreover, sequences belonging to other countries and of zero-distance to our isolates were shown in bold and clustered.

**Table S2. Best fitting model selection using Maximum Likelihood fits of 24 different nucleotide substitution models**

| **Model** | **#Param** | **BIC** | **AICc** | **lnL** | ***I*** | ***G*** | **R** | **Freq A** | **Freq T** | **Freq C** | **Freq G** | **A=>T** | **A=>C** | **A=>G** | **T=>A** | **T=>C** | **T=>G** | **C=>A** | **C=>T** | **C=>G** | **G=>A** | **G=>T** | **G=>C** |
| --- | --- | --- | --- | --- | --- | --- | --- | --- | --- | --- | --- | --- | --- | --- | --- | --- | --- | --- | --- | --- | --- | --- | --- |
| **T92+I*** | 188 | **3624.7** | 2182.3 | -900.9 | 0.45 | n/a | 1.82 | 0.34 | 0.34 | 0.16 | 0.16 | 0.06 | 0.03 | 0.11 | 0.06 | 0.11 | 0.03 | 0.06 | 0.23 | 0.03 | 0.23 | 0.06 | 0.03 |
| T92+G | 188 | 3630.9 | 2188.4 | -904.0 | n/a | 0.56 | 1.91 | 0.34 | 0.34 | 0.16 | 0.16 | 0.05 | 0.02 | 0.11 | 0.05 | 0.11 | 0.02 | 0.05 | 0.24 | 0.02 | 0.24 | 0.05 | 0.02 |
| T92+G+I | 189 | 3634.1 | 2184.0 | -900.8 | 0.42 | 6.52 | 1.85 | 0.34 | 0.34 | 0.16 | 0.16 | 0.05 | 0.02 | 0.11 | 0.05 | 0.11 | 0.02 | 0.05 | 0.23 | 0.02 | 0.23 | 0.05 | 0.02 |
| HKY+I | 190 | 3639.4 | 2181.6 | -898.6 | 0.45 | n/a | 1.82 | 0.37 | 0.32 | 0.13 | 0.18 | 0.05 | 0.02 | 0.12 | 0.06 | 0.09 | 0.03 | 0.06 | 0.22 | 0.03 | 0.25 | 0.05 | 0.02 |
| HKY+G | 190 | 3641.5 | 2183.8 | -899.6 | n/a | 0.55 | 1.90 | 0.37 | 0.32 | 0.13 | 0.18 | 0.05 | 0.02 | 0.13 | 0.06 | 0.09 | 0.03 | 0.06 | 0.22 | 0.03 | 0.25 | 0.05 | 0.02 |
| TN93+I | 191 | 3646.8 | 2181.4 | -897.4 | 0.45 | n/a | 1.88 | 0.37 | 0.32 | 0.13 | 0.18 | 0.05 | 0.02 | 0.15 | 0.06 | 0.07 | 0.03 | 0.06 | 0.17 | 0.03 | 0.29 | 0.05 | 0.02 |
| HKY+G+I | 191 | 3648.8 | 2183.4 | -898.4 | 0.42 | 6.53 | 1.85 | 0.37 | 0.32 | 0.13 | 0.18 | 0.05 | 0.02 | 0.12 | 0.06 | 0.09 | 0.03 | 0.06 | 0.22 | 0.03 | 0.25 | 0.05 | 0.02 |
| TN93+G | 191 | 3654.8 | 2189.4 | -901.4 | n/a | 0.56 | 1.95 | 0.37 | 0.32 | 0.13 | 0.18 | 0.05 | 0.02 | 0.14 | 0.06 | 0.07 | 0.03 | 0.06 | 0.18 | 0.03 | 0.29 | 0.05 | 0.02 |
| T92 | 187 | 3655.8 | 2221.0 | -921.3 | n/a | n/a | 1.59 | 0.34 | 0.34 | 0.16 | 0.16 | 0.06 | 0.03 | 0.1 | 0.06 | 0.1 | 0.03 | 0.06 | 0.22 | 0.03 | 0.22 | 0.06 | 0.03 |
| TN93+G+I | 192 | 3656.5 | 2183.4 | -897.4 | 0.44 | 14.66 | 1.90 | 0.37 | 0.32 | 0.13 | 0.18 | 0.05 | 0.02 | 0.15 | 0.06 | 0.07 | 0.03 | 0.06 | 0.17 | 0.03 | 0.29 | 0.05 | 0.02 |
| K2+I | 187 | 3662.7 | 2227.9 | -924.8 | 0.46 | n/a | 1.69 | 0.25 | 0.25 | 0.25 | 0.25 | 0.05 | 0.05 | 0.16 | 0.05 | 0.16 | 0.05 | 0.05 | 0.16 | 0.05 | 0.16 | 0.05 | 0.05 |
| K2+G | 187 | 3668.6 | 2233.8 | -927.7 | n/a | 0.59 | 1.73 | 0.25 | 0.25 | 0.25 | 0.25 | 0.05 | 0.05 | 0.16 | 0.05 | 0.16 | 0.05 | 0.05 | 0.16 | 0.05 | 0.16 | 0.05 | 0.05 |
| GTR+I | 194 | 3670.5 | 2182.2 | -894.7 | 0.44 | n/a | 1.73 | 0.37 | 0.32 | 0.13 | 0.18 | 0.04 | 0.04 | 0.13 | 0.04 | 0.07 | 0.03 | 0.11 | 0.17 | 0.03 | 0.27 | 0.05 | 0.02 |
| HKY | 189 | 3670.6 | 2220.5 | -919.0 | n/a | n/a | 1.58 | 0.37 | 0.32 | 0.13 | 0.18 | 0.06 | 0.02 | 0.12 | 0.07 | 0.08 | 0.03 | 0.07 | 0.21 | 0.03 | 0.24 | 0.06 | 0.02 |
| K2+G+I | 188 | 3670.6 | 2228.2 | -923.9 | 0.45 | 39.53 | 1.69 | 0.25 | 0.25 | 0.25 | 0.25 | 0.05 | 0.05 | 0.16 | 0.05 | 0.16 | 0.05 | 0.05 | 0.16 | 0.05 | 0.16 | 0.05 | 0.05 |
| GTR+G | 194 | 3672.4 | 2184.1 | -895.7 | n/a | 0.55 | 1.82 | 0.37 | 0.32 | 0.13 | 0.18 | 0.03 | 0.04 | 0.13 | 0.04 | 0.08 | 0.03 | 0.12 | 0.19 | 0.03 | 0.25 | 0.05 | 0.02 |
| GTR+G+I | 195 | 3679.1 | 2183.1 | -894.2 | 0.42 | 6.84 | 1.81 | 0.37 | 0.32 | 0.13 | 0.18 | 0.03 | 0.04 | 0.14 | 0.04 | 0.07 | 0.03 | 0.11 | 0.18 | 0.03 | 0.27 | 0.05 | 0.02 |
| TN93 | 190 | 3681.7 | 2223.9 | -919.7 | n/a | n/a | 1.58 | 0.37 | 0.32 | 0.13 | 0.18 | 0.06 | 0.02 | 0.13 | 0.07 | 0.08 | 0.03 | 0.07 | 0.19 | 0.03 | 0.25 | 0.06 | 0.02 |
| K2 | 186 | 3690.5 | 2263.3 | -943.5 | n/a | n/a | 1.53 | 0.25 | 0.25 | 0.25 | 0.25 | 0.05 | 0.05 | 0.15 | 0.05 | 0.15 | 0.05 | 0.05 | 0.15 | 0.05 | 0.15 | 0.05 | 0.05 |
| JC+I | 186 | 3692.7 | 2265.6 | -944.6 | 0.45 | n/a | 0.5 | 0.25 | 0.25 | 0.25 | 0.25 | 0.08 | 0.08 | 0.08 | 0.08 | 0.08 | 0.08 | 0.08 | 0.08 | 0.08 | 0.08 | 0.08 | 0.08 |
| JC+G | 186 | 3696.8 | 2269.7 | -946.7 | n/a | 0.64 | 0.5 | 0.25 | 0.25 | 0.25 | 0.25 | 0.08 | 0.08 | 0.08 | 0.08 | 0.08 | 0.08 | 0.08 | 0.08 | 0.08 | 0.08 | 0.08 | 0.08 |
| JC+G+I | 187 | 3702.4 | 2267.6 | -944.6 | 0.45 | 200 | 0.5 | 0.25 | 0.25 | 0.25 | 0.25 | 0.08 | 0.08 | 0.08 | 0.08 | 0.08 | 0.08 | 0.08 | 0.08 | 0.08 | 0.08 | 0.08 | 0.08 |
| GTR | 193 | 3703.2 | 2222.5 | -915.9 | n/a | n/a | 1.48 | 0.37 | 0.32 | 0.13 | 0.18 | 0.05 | 0.04 | 0.12 | 0.05 | 0.07 | 0.03 | 0.12 | 0.19 | 0.03 | 0.23 | 0.05 | 0.02 |
| JC | 185 | 3719.4 | 2299.9 | -962.8 | n/a | n/a | 0.5 | 0.25 | 0.25 | 0.25 | 0.25 | 0.08 | 0.08 | 0.08 | 0.08 | 0.08 | 0.08 | 0.08 | 0.08 | 0.08 | 0.08 | 0.08 | 0.08 |

*Models with the lowest BIC scores (Bayesian Information Criterion) are considered to describe the substitution pattern the best (written in red and bold). For each model, AICc value (Akaike Information Criterion, corrected), Maximum Likelihood value (lnL), and the number of parameters (including branch lengths) are also depicted above. 5 rate categories-discrete Gamma distribution (+*G*) was applied for modeling the evolutionary rates non-uniformity among sites and supposing that a certain fraction of sites is evolutionarily invariable (+*I*). Proposed or estimated values of transition/transversion bias (R) for each model are displayed. They are followed by nucleotide frequencies (f) and rates of base substitutions (r) for each nucleotide pair. A total of 173 positions existed in the final dataset. Abbreviations: TR: General Time Reversible; HKY: Hasegawa-Kishino-Yano; TN93: Tamura-Nei; T92: Tamura 3-parameter; K2: Kimura 2-parameter; JC: Jukes-Cantor.

**Table S3. Accession numbers of sequences used for phylogenetic analysis of human rotavirus A isolates**

| **Accession number** | **Abbreviated sequence name*** |
| --- | --- |
| LC500701 | RVA/Human/JPN/BLU5-vp7/2018/G2P4 |
| MN621367 | RVA/Sew/SP/R16-vp7/2017/G2P8 |
| MN621366 | RVA/Sew/SP/R12A-vp7/2016/G2P8 |
| MT053458 | RVA/Human-wt/GER/FB041/2017/G2P4 |
| MK873107 | RVA/Human-wt/THA/SiRAV-667/2017/G2P8 |
| MH591276 | RVA/Human-wt/LBNH179/2012/G2P8 |
| MH591270 | RVA/Human-wt/LBNM147/2011/G2P4 |
| MN066795 | RVA/Human-wt/IND/CMC_00025/2012/G2P8 |
| MG652327 | RVA/Human-wt/DOM/3000503734/2016/G2P8 |
| MK814561 | RVA/Human-wt/IDN/YK-RVY47/2009/G2P4 |
| MH382856 | RVA/Human-wt/ETH/BD522/2016/G2P4 |
| MG996103 | RVA/Human-wt/SGP/NV-16-161/2016/G2P4 |
| MH625736 | RVA/Human-wt/CHN/G12021182/2012/G2P4 |
| HQ537515 | RVA/Human-wt/RUS/Nov10-N397/2010/G2P4 |
| MG181826 | RVA/Human-wt/MWI/BID11E/2012/G2P4 |
| KY489874 | RVA/Human-wt/TWN/07-96s-498/2007/G2P4 |
| MN478499 | RVA/Human-wt/USA/2014741096/2014/G2P4 |
| LC328924 | RVA/Human-wt/JPN/HC10062/2010/G2P4 |
| MN856459 | RVA/Human-wt/CHN/G17081040/2017/G2P4 |
| MN836878 | RVA/Human/THA/B4285/2017/G2P8 |
| MN552044 | RVA/Human-wt/RUS/NS16-A415/2016/G2P4 |
| MH182474 | RVA/Human-wt/PAK/PAK205/2015/G2P4 |
| MH557060 | RVA/Human-wt/PAK/NIH-BBH-4705/2015/G2P4 |
| MF673441 | RVA/Human-wt/PAK/NIH-KGH-4455/2016/G2P4 |
| KU532216 | RVA/Human-wt/CHN/SH-RV76/2015/G2P4 |
| KF812583 | RVA/Human/KOR/Seoul1433/2010/G2P4 |
| KJ870821 | RVA/Human-wt/COD/KisB523/2009/G2P4 |
| KF006866 | RVA/Human/RUS/O1270/2011/G2P8 |
| JX841121 | RVA/Human/RUS/Nov11-N1936/2011/G2P8 |
| JX156396 | RVA/Human/RUS/O1157/2011/G2P4 |
| MN837061 | RVA/Human/THA/B6295/2018/G2P8 |
| MN837033 | RVA/Human/THA/B5613/2018/G2P4 |
| MN577199 | RVA/Human-wt/RUS/NS18-A1455/2018/G2P4 |
| KR705272 | RVA/Human-wt/BEL/BE34/2006/G2P4 |
| KF648941 | RVA/Human/RUS/S12-14/2012/G2P4 |
| JQ069504 | RVA/Human-wt/CAN/RT125-07/2008/G2P4 |
| AB821344 | RVA/Human-wt/JPN/B110056/2011/G2P4 |
| KC443768 | RVA/Human-wt/AUS/CK20027/2006/G2P4 |
| JQ837882 | RVA/Human-wt/IDN/BL-5210/2006/G2P4 |
| JX273718 | RVA/Human-wt/PAK-HF56/2010/G2P4 |
| MW058329 | RVA/Human/THA/B5581/2018/G2P8 |
| MT710809 | RVA/Human-wt/CHN/SX/2018/393/G2P4 |
| KP281273 | RVA/Human/SA/Taif-1/2013/G1P8 |
| KP281274 | RVA/Human/SA/Taif-2/2013/G1P8 |
| KP281275 | RVA/Human/SA/Taif-3/2013/G1P8 |
| KP281276 | RVA/Human/SA/Taif-4/2013/G1P8 |
| KP281277 | RVA/Human/SA/Taif-5/2013/G1P8 |
| KT257308 | RVA/Human-wt/LBN/N318/2012/G1P8 |
| MH591261 | RVA/Human-wt/LBN/A167/2013/G1P8 |
| AB297791 | RVA/Human/SA/MD28/2007/G9P8 |
| MH591313 | RVA/Human-wt/LBN/H256/2013/G9P8 |
| MH591314 | RVA/Human-wt/LBNA152/2012/G9P8 |
| MN061928 | RVA/Human-wt/TUN/268/2017/G9P8 |
| MN061926 | RVA/Human-wt/TUN/511/2016/G9P8 |
| JN001866 | RVA/human-wt/PAK/NIBGE-59/2010/G9P8 |
| EF179200 | RVA/Human/PAR/Py02AP2/2005/G9P8 |
| KF414571 | RVA/Human-wt/ITA/SS54/2011/G9P8 |
| KJ454498 | RVA/Human-wt/BRA/MA20306/2011/G9P8 |
| KT920656 | RVA/Human-wt/USA/CNMC122/2011/G12P8 {Outgroup} |

*Abbreviated sequence name followed the order of Virus_name/Source/Country_of_origin/Isolate_name/Collection_date/Genotype.
